# Supplementary material for: Rapa Nui (Easter Island) Rano Raraku crater lake basin: Geochemical characterization and implications for the Ahu-Moai Period
Source: PLoS One. 2021 Oct 13;16(10):e0254793. doi: 10.1371/journal.pone.0254793 (PMC8513918; doi:10.1371/journal.pone.0254793)
Supplement: Attachment — Submitted filename: PONE-D-20-39268_reviewer.pdf [file pone.0254793.s001.pdf]

PLOS ONE

Easter Island Rano Raraku crater lake basin: geochemical characterization and implications for the Moai cult society

--Manuscript Draft--

**Manuscript Number:**	PONE-D-20-39268
**Article Type:**	Research Article
**Full Title:**	Easter Island Rano Raraku crater lake basin: geochemical characterization and implications for the Moai cult society
**Short Title:**	Geochemical Characterization of the Rano Raraku (Rapa Nui/Easter Island) Crater Lake Basin
**Corresponding Author:**	Mara Bortolini<br>Ca'Foscari University of Venice: Universita Ca' Foscari<br>Venezia, VE ITALY
**Keywords:**	Easter Island; Rapa Nui; Rano Raraku; LREE-HREE; oxic/anoxic conditions; lake sediments; water supply
**Abstract:**	Rano Raraku, the crater lake constrained by basaltic tuffs that served as the primary quarry for the moai statues on Rapa Nui (Easter Island), has experienced fluctuations in lake level over the past centuries. As one of the only freshwater sources on the island, understanding the present and past geochemical characteristics of the lake water is critical to understand if it could have been a viable fresh water source, as well as better understanding past human-environment interactions. At the time of sampling in September 2017, lake maximum depth was 1.5 m. The lake level has substantially declined in the subsequent years, with the lake drying almost completely in January 2018. The lake is currently characterized by highly anoxic conditions, with a predominance of ammonium ions on nitrates, a high concentration of organic carbon in the water-sediment interface and reducing conditions of the lake, as evidenced by Mn/Fe and Cr/V ratios. The estimate of past salinity inferred from the chloride mass balance shows how the basin was likely not considered as a main freshwater source by early Rapa Nui people.
**Order of Authors:**	E. Argiriadis
	M. Bortolini
	N.M. Kehrwald
	M. Roman
	C. Turetta
	S. Hanif
	E.O. Erhenhi
	J.M. Ramirez Aliaga
	D. B. McWethy
	A. E. Myrbo
	A. Pauchard
	C. Barbante
	D. Battistel
**Additional Information:**	
**Question**	**Response**
**Financial Disclosure**<br><br>Enter a financial disclosure statement that	The authors received no specific funding for this work.

Powered by Editorial Manager® and ProduXion Manager® from Aries Systems Corporation

describes the sources of funding for the work included in this submission. Review the submission guidelines for detailed requirements. View published research articles from *PLOS ONE* for specific examples.<br><br>This statement is required for submission and **will appear in the published article** if the submission is accepted. Please make sure it is accurate.<br><br>**Unfunded studies**<br>Enter: *The author(s) received no specific funding for this work.*<br><br>**Funded studies**<br>Enter a statement with the following details:<br>• Initials of the authors who received each award<br>• Grant numbers awarded to each author<br>• The full name of each funder<br>• URL of each funder website<br>• Did the sponsors or funders play any role in the study design, data collection and analysis, decision to publish, or preparation of the manuscript?<br>• **NO** - Include this sentence at the end of your statement: *The funders had no role in study design, data collection and analysis, decision to publish, or preparation of the manuscript.*<br>• **YES** - Specify the role(s) played.<br><br>* typeset	
**Competing Interests**<br><br>Use the instructions below to enter a competing interest statement for this submission. On behalf of all authors, disclose any competing interests that could be perceived to bias this work—acknowledging all financial support and any other relevant financial or non-financial competing interests.<br><br>This statement **will appear in the published article** if the submission is accepted. Please make sure it is accurate. View published research articles from *PLOS ONE* for specific examples.	The authors have declared that no competing interests exist.

Powered by Editorial Manager® and ProduXion Manager® from Aries Systems Corporation

**NO authors have competing interests**<br><br>Enter: *The authors have declared that no competing interests exist.*<br><br>**Authors with competing interests**<br><br>Enter competing interest details beginning with this statement:<br><br>*I have read the journal's policy and the authors of this manuscript have the following competing interests: [insert competing interests here]*<br><br>* typeset	
**Ethics Statement**<br><br>Enter an ethics statement for this submission. This statement is required if the study involved:<br><br>• Human participants<br>• Human specimens or tissue<br>• Vertebrate animals or cephalopods<br>• Vertebrate embryos or tissues<br>• Field research<br><br>Write "N/A" if the submission does not require an ethics statement.<br><br>General guidance is provided below. Consult the submission guidelines for detailed instructions. **Make sure that all information entered here is included in the Methods section of the manuscript.**	N/A

**Format for specific study types**<br><br>**Human Subject Research (involving human participants and/or tissue)**<br>• Give the name of the institutional review board or ethics committee that approved the study<br>• Include the approval number and/or a statement indicating approval of this research<br>• Indicate the form of consent obtained (written/oral) or the reason that consent was not obtained (e.g. the data were analyzed anonymously)<br><br>**Animal Research (involving vertebrate animals, embryos or tissues)**<br>• Provide the name of the Institutional Animal Care and Use Committee (IACUC) or other relevant ethics board that reviewed the study protocol, and indicate whether they approved this research or granted a formal waiver of ethical approval<br>• Include an approval number if one was obtained<br>• If the study involved *non-human primates*, add *additional details* about animal welfare and steps taken to ameliorate suffering<br>• If anesthesia, euthanasia, or any kind of animal sacrifice is part of the study, include briefly which substances and/or methods were applied<br><br>**Field Research**<br><br>Include the following details if this study involves the collection of plant, animal, or other materials from a natural setting:<br>• Field permit number<br>• Name of the institution or relevant body that granted permission	
**Data Availability**<br><br>Authors are required to make all data underlying the findings described fully available, without restriction, and from the time of publication. PLOS allows rare exceptions to address legal and ethical concerns. See the PLOS Data Policy and FAQ for detailed information.	Yes - all data are fully available without restriction

A Data Availability Statement describing where the data can be found is required at submission. Your answers to this question constitute the Data Availability Statement and **will be published in the article**, if accepted.<br><br>**Important:** Stating 'data available on request from the author' is not sufficient. If your data are only available upon request, select 'No' for the first question and explain your exceptional situation in the text box.<br><br>Do the authors confirm that all data underlying the findings described in their manuscript are fully available without restriction?	
**Describe where the data may be found in full sentences. If you are copying our sample text, replace any instances of XXX with the appropriate details.**<br><br>• If the data are **held or will be held in a public repository**, include URLs, accession numbers or DOIs. If this information will only be available after acceptance, indicate this by ticking the box below. For example: *All XXX files are available from the XXX database (accession number(s) XXX, XXX.).*<br>• If the data are all contained **within the manuscript and/or Supporting Information files**, enter the following: *All relevant data are within the manuscript and its Supporting Information files.*<br>• If neither of these applies but you are able to provide **details of access elsewhere**, with or without limitations, please do so. For example:<br><br>*Data cannot be shared publicly because of [XXX]. Data are available from the XXX Institutional Data Access / Ethics Committee (contact via XXX) for researchers who meet the criteria for access to confidential data.*<br><br>*The data underlying the results presented in the study are available from (include the name of the third party*	All the data collected for this research are available on Mendeley Data using the following link https://data.mendeley.com/datasets/k93rp3p4pd/1.

and contact information or URL).<br>• This text is appropriate if the data are owned by a third party and authors do not have permission to share the data.<br><br>* typeset	
Additional data availability information:	

Manuscript Click here to access/download;Manuscript;Manuscript.docx

Easter Island Rano Raraku crater lake basin: geochemical characterization and implications for the Moai cult society

E. Argiriadis[1,2], M. Bortolini[2,*], N.M. Kehrwald[3], M. Roman[2], C. Turetta[1], S. Hanif[2], E.O. Erhenhi[2], J.M. Ramirez Aliaga[4], D. B. McWethy[5], A. E. Myrbo[6], A. Pauchard[7], C. Barbante[1,2], D. Battistel[1,2]

[1]Institute of Polar Sciences CNR-ISP, via Torino 155, 30172 Venice, Italy

[2]Department of Environmental Sciences, Informatics and Statistics, Ca' Foscari University of Venice, Via Torino 155, 30172, Venice, Italy

[3]U.S. Geological Survey, Geosciences and Environmental Change Science Center, MS 980, Denver, CO, 80225, USA

[4]Centro de Estudios Avanzados, Universidad de Playa Ancha, Traslaviña 450, Viña del Mar, Chile

[5]Department of Earth Sciences, Montana State University, Bozeman, MT, United States.

[6]Department of Earth Sciences, University of Minnesota, Twin Cities, Minneapolis, MN, United States

[7]Laboratorio de Invasiones Biológicas, Facultad de Ciencias Forestales, Universidad de Concepción, Concepción, Chile

* Corresponding author e-mail: mara.bortolini@unive.it

Abstract

Rano Raraku, the crater lake constrained by basaltic tuffs that served as the primary quarry for the *moai* statues on Rapa Nui (Easter Island), has experienced fluctuations in lake level over the past centuries. As one of the only freshwater sources on the island, understanding the present and past geochemical characteristics of the lake water is critical to understand if it could have been a viable fresh water source, as well as better understanding past human-environment interactions. At the time of sampling in September 2017, lake maximum depth was 1.5 m. The lake level has substantially declined in the subsequent years, with the lake drying almost completely in January 2018. The lake is currently characterized by highly anoxic conditions, with a predominance of ammonium ions on nitrates, a high concentration of organic carbon in the water-sediment interface and reducing conditions of the lake, as evidenced by Mn/Fe and Cr/V ratios. The estimate of past salinity inferred from the chloride mass balance shows how the basin was likely not considered as a main freshwater source by early Rapa Nui people.

The installation of an outlet pipe around 1950 and active until the late 1970s, as well as grazing of horses on the lake margins appear to have impacted significantly on the geochemical conditions of Rano Raraku sediments and lake water in recent decades. Such impacts are well distinct from natural environmental changes and highlight the need to consider the sensitivity of the lake geochemistry to human activities.

Keywords: Easter Island, Rapa Nui, Rano Raraku, LREE-HREE, oxic/anoxic conditions, lake sediments, water supply.

Introduction

Hundreds of massive megalithic statues, known as *moai*, populate the landscape of Rapa Nui (Easter Island). The crater walls constraining Rano Raraku served as the primary quarry for *moai* by the ancient Rapa Nui inhabitants between ~1200 and 1600 CE (Hamilton et al., 2011). Due to the importance of Rano Raraku to the creation of the *moai*, archeologists have intensely investigated this site (Dumont et al. 1998; Azizi and Flenley, 2008; Mann at al., 2008; Saez at al., 2009; Rull at al., 2010; Cañellas-Boltà et al., 2012, 2013, 2016; Horrocks et al., 2012). These studies demonstrate a consensus on the cultural centrality of Rano Raraku from ~ 1200 until at least ~ 1450 CE. During the time when humans were carving *moai*, people cultivated taro and sweet potato in adjacent uplands (Horrocks et al., 2012), and palms were widespread in the landscape surrounding Rano Raraku (Cañellas-Boltà et al., 2013). The timing and reasons for the cessation of *moai* quarrying at Rano Raraku are hotly debated. Many explanations suggest abandonment of quarrying may have been associated with major cultural and environmental changes on the island (Dumont et al., 1998; Flenley and Bahn, 2003; Diamond, 2005; Nunn, 2007; Lipo et al., 2016; Rull, 2016; Rull et al., 2018).

The Rano Raraku crater was formed ~ 0.7 mya along the Rano Kau ridge. It is a single tuff cone, and in some sectors, the tuff is strongly altered in reddish ash. The tuff is stratified, composed of sideromelane, slightly altered to palagonite, and contains lithic blocks of older lavas. Scoria-ceous basaltic lapilli are present with of olivine, clinopyroxene, plagioclase, and ilmenite (Vezzoli and Acocella, 2009).

The crater catchment supports a lake (~ 80 masl) which is directly adjacent to one of the primary moai quarries on Rapa Nui, providing a link between archeological investigations and evidence of past environmental changes. Rano Raraku (~ 300 m in diameter) has a small catchment

size on the scale of ~ 1100 m^2, is a closed basin with no inflow or outflow streams and is therefore an ideal archive for paleoenvironmental reconstructions (Cañellas-Boltà et al. 2013, Flenley and Bahn, 2003; Flenley, 1993, 1996; Flenley and King, 1984).

Lake sediments contain multiple proxies for reconstructing past environments and geochemical conditions (e.g., charcoal, pollen, biological indicators and elemental chemistry; Barker et al., 1994; Dean et al., 2015; Ito, 2002). Additionally, lake level fluctuations, for example, can be reconstructed through marginal depositional facies (Magny, 2006). Past variability in salinity can be inferred from Mg/Ca and Ca/Sr ratios. In more saline water, the precipitation of high-Mg aragonite (or calcite) is favored compared to low-Mg calcite, resulting in an increased Mg/Ca ratio in the sediment. As aragonite is also enriched in Sr, the Mg/Ca increase generally coincides with a Ca/Sr decrease (Kelts and Hsü,1978; Tesoriero and Pankow, 1996; Ito, 2002; Dissard et al., 2010). Lakes respond dynamically to changes in the water balance, especially in closed systems. When evaporation increases, lake water becomes more saline and its composition becomes enriched in the heavier oxygen and hydrogen isotopes ($\delta^{18}O$ and $\delta^{2}H$) (Leng and Marshall, 2004).

Lake conditions also affect the ratios of several redox sensitive elements, such as Mn/Fe (Naeher et al., 2013) and Cr/V (Schaller et al., 1997). Changes in the oxidation state of Manganese (Mn) and Iron (Fe) affect their solubility and mobility. Mn and Fe have different redox potentials, so that Mn is reduced (oxidized) faster (slower) than Fe, resulting in a decrease in Mn/Fe in the sediment when oxygen concentrations decrease in the water column (Davison et al., 1982; Davison 1993; Martin 2005). Chromium (Cr) and Vanadium (V) exhibit a similar behavior, with distinct oxidation states as well as the tendency to form complexes with Fe-hydroxides or organic ligands, depending on their redox state (Wehrli and Stumm, 1989). As a result, Cr/V increases in sediments under anoxic conditions, and decreases in oxic conditions. Rare earth elements (REEs) help determine past

hydrological and transport processes inside the basin, lake level variability and *in situ* partitioning (Battistel et al., 2018). The fractionation of light and heavier rare earth elements (LREEs and HREEs, respectively) is promoted by preferential leaching during runoff and/or the formation of compounds with different solubilities in the aquatic environment, as HREE complexes are generally more soluble than the corresponding LREEs (Aide and Aide, 2012). The different forms of the REE cerium can provide additional information on fractionation processes and redox conditions. Both of the oxidized forms of Ce (trivalent and tetravalent) are less soluble than the other REEs. Therefore, leaching processes can lead to fractionation because more soluble Ce species are preferentially transported into the lake where redox condition drives the mobility cycle of cerium, in oxic conditions Ce(IV) prevails resulting in the enrichment of the element in the sediment and subsequent depletion in the lake water (Holser, 1997; Xiong et al., 2012).

In this paper, we investigate the modern environmental processes at Rano Raraku as recorded from trace and rare earth elements in water, soil, and surface sediments and their application for past environmental changes. This study expands upon studies of major elements (Si, Ca, S, Fe and Ti) (Saez et al., 2009), pH and electrical conductivity (Geller, 1992) and macro and microfossils in a soil survey (Horrocks et al., 2012). This study provides a detailed characterization of modern lake and geochemical conditions that can be used to enrich interpretations of past environmental change from paleoecological records from Rano Raraku. Moreover, the reconstruction of the lake conditions can help elucidate some important questions about the freshwater availability in the island and the cultural shift from the Ancient to the Birdman Cult.

1. Materials and Methods

2.1 Study Site

Rapa Nui (Easter Island) is a small island that rises above sea level (~160 km^2) from a large volcanic complex located in the Pacific Ocean (27°09'S, 109°26'W) (fig. 1). Rapa Nui is one of the most isolated inhabited islands in the world as the nearest mainland is the Chilean coast, more than 3500 km away. The climate is warm-temperate, with remarkably stable seasonal and daily temperatures ranging from mean ~ 18°C in July-September to mean ~ 23°C in January-March. Mean annual precipitation is ~ 1070 mm, and monthly precipitation amounts are slightly more abundant in April-June (mean monthly values > 100 mm) than other months of the year. The rainfall distribution significantly differs across the island, with higher precipitation at higher elevations (e.g., Mount Terevaka, 507 masl) and lower precipitation in lowlands including Hanga Roa (12 masl), the primary town on Rapa Nui (Herrera and Custodio, 2008). Rainfall quickly infiltrates volcanic soils on Rapa Nui and, as a result, few streams or surface water bodies exist on the island. Rano Raraku (fig. 1) is one of the three main freshwater sources of the island along with Rano Kau and Rano Aroi. The crater is > 300,000 years old (Baker et al., 1974) and consists of basaltic lapilli tuff used in the statues (Gonzalez-Ferran and Baker, 1974). The chemistry and petrography of basaltic rocks and tuff of the Rano Raraku quarry showed a general enrichment in Fe and a depletion of alkali and silica content of ~55-60 % (Gioncada et al. 2010; Baker et al. 1974).

Large-scale climatic variability has acted as a primary control on lake level and sedimentation dynamics during the last 34,000 years (Saez et al., 2009; Cañellas-Boltà et al., 2012). Increased sedimentation of material largely derived from the catchment indicates strong erosion and runoff around the termination of the last Glacial (34,000-14,600 cal. yr BP, Cañellas-Boltà et al., 2012). The Early Holocene was characterized by warmer conditions, reduced runoff and increased productivity, while in the Mid-Holocene (8700-4500 cal. yr BP) the lake level decreased significantly, promoting

the onset of anoxic conditions. The lake dried leading to hiatuses in sedimentation between 4500 and 800 cal. yr BP. Sedimentation resumed after 800 cal. yr BP. However, drying may have occurred many times in the recent decades to centuries, as a result of changes in land use and the introduction of semi-wild sheep, cattle and horses around 1864 CE, where their grazing and associated erosion impacted the hydrological system.

Figure 1. (a) The location of Rano Raraku on Rapa Nui (Easter Island). (b) Aerial view of the crater lake with the positions of the samples collected. (c) Picture of the Rano Raraku basin on 7 Sep 2017.

2.2 Sample collection

Three surface water samples (~ 500 mL) were collected at P1, P2 and P3 in September 2017 (fig. 1). At each of the three locations, P1-P3, water samples (~500 mL) were also collected from the water column at depths of 90 (P1), 51 (P2) and 38 (P3) cm, respectively. Six 4-cm thick topsoil samples (~5 g) were collected at P4, P5, P6 and P7 (fig. 1). Exact locations of the sampling points are reported in fig. 1 and table 1 records the characteristics of each sample. A lake sediment core (LTS-RKU17-1A-1P-1), approximately 1 m long, was collected from 95 cm below the water surface using a Livingstone-type surface corer (Wright, 1967) at -27.12220, -109.29031. In this paper, we examine sediment samples corresponding to (a) the water-sediment interface (WSI; top 2 cm) and (b) bed sediments (between 3-5 cm depth).

Table 1. Characteristics of sample collected at Rano Raraku.

Location	Samples	Depth (cm)	Elevation (masl)	Volume / Weight
P1	Water – Suspended particulate matter (SPM)	0	-	500 ml
	Deep water – SPM	90	-	500 ml
	Water-sediment interface (WSI)	0-2	-	5 g
	Bed sediment	3-5	-	5 g
P2	Water – SPM	0	-	500 ml
	Deep water – SPM	50	-	500 ml
P3	Water – SPM	0	-	500 ml
	Deep water – SPM	38	-	500 ml
P4	Topsoil	-	82	5 g
P5	Topsoil	-	88.5	5 g
	Topsoil		89	5 g
P6	Topsoil	-	80	5 g
	Topsoil		81	5 g
P7	Topsoil	-	105	5 g

2.3 Sample Preparation and Analysis

All water samples were filtered *in situ* through 0.45 µm cellulose nitrate membranes (Whatman) using a polycarbonate system equipped with a manual vacuum pump. Membranes containing suspended particulate matter (SPM) were stored in plastic Petri dishes, while the filtered water was kept in 50 mL HDPE bottles. All samples were stored at 4 °C before being shipped to the Ca' Foscari University of Venice for analysis. Soil and sediment samples were dried to a constant weight in a desiccator at room temperature before further preparation for the analysis.

2.4 Water analysis

Major ions (Cl^-, SO_4^{2-}, NO_3^-, NH_4^+, Na^+, K^+, Ca^{2+} and Mg^{2+}) in water samples were analyzed using an ion chromatography system (883 Basic IC plus, Metrohm) equipped with a Metrosep Cation 1-2 (particle size 7 µm; eluent: HNO_3 3 mM) and a Metrosep Anion supp/4 (particle size 5 µm; eluent: HCO_3^-/CO_3^{2-} buffer 1.7/1.8 mM) column for cation and anion analysis, respectively. Liquid samples were directly injected into the chromatographic system after filtration through 0.45 µm syringe filters. Quantification of trace elements (TEs) and REEs in water samples were performed at the ISP-CNR laboratory in Venice (Italy) using an inductively coupled plasma - sector field mass spectrometer (ICP-SFMS model Element-XR, Thermo Scientific, Bremen, Germany). Water samples were acidified with ultrapure grade HNO_3 (Romil) and analyzed 24 hours later. TEs and REEs were quantified using external calibration curves. Method accuracy was assessed through the analysis of a certified reference material (TMRAIN95).

The isotopic composition of water samples was determined with a DeltaV-Advantage mass spectrometer (Thermo Scientific) equipped with a gas-bench. For the analysis, 200 µL of water sample were introduced into a glass vial. The samples were first flushed with a gas mixture of 2 % H_2 in He and analyzed to determine δ^2H. The same samples were then flushed with a gas mixture of 0.4 % CO_2 in He and analyzed to determine $\delta^{18}O$ after 20 hours of equilibration. All samples were measured at least in triplicate. The SMOW2 and SLAP2 isotopic standards were used as references together with a laboratory standard that was analysed every 9 samples to evaluate the stability of measurements. The isotopic composition is expressed as $\delta\ R = [(R_s - R_r)/R_r] \times 1000$ where δR is δ^2H or $\delta^{18}O$, R is $^2H/H$ or $^{18}O/^{16}O$ in the sample (s) and in the reference (r) respectively.

2.5 Sediment, soil and SPM analysis

Sediment, soil and SPM samples were digested with a mixture of HNO_3, HCl and HF (6:2:1 mL for 0.2 g of sample, suprapure grade acids, Romil), using an Ethos 1 microwave oven (Milestone) and a temperature-controlled program up to 200 °C in pressurized Teflon vessels. The digests were diluted in 50 mL of ultra-pure water, randomized and analyzed within 24 hours for determination of major, trace and rare earth elements. The analysis was carried out by ICP-MS (iCAP RQ, Thermo Scientific) equipped with an ASX-560 autosampler (Teledyne Cetac Technologies), PFE cyclonic spray chamber thermostatted at 2.7 °C, sapphire injector, quartz torch, Ni cones and 1550 W of plasma RF power. Triplicate acquisitions were performed in kinetic energy discrimination (KED) – high matrix mode (collision gas He). Quantification was obtained by external calibration with standard prepared by mixing the multi-elemental solutions IMS-101, IMS-102 and IMS-104 (UltraScientific), and using on-line spiked Rh as the internal standard. Accuracy was assessed by contextual mineralization and analysis of the certified reference materials NIMT/UOE/FM/001 (peat) and BCR-667 (estuarine sediment).

Total Organic Carbon in sediments (TOC %) was determined with a Flash 2000 HT Elemental Analyzer (Thermo Scientific). A small amount of dry sample (~ 0.2 mg) was weighed and sealed into tin capsules, then introduced into the CHNO quartz reactor, composed of chromium oxide, copper and silvered cobalt, and cobalt oxide. The reactor temperature was set at 950 °C, while the column was held at 65 °C. Samples were analyzed in triplicate.

2. Results and Discussion

3.1 Source of the Rano Raraku water

The absence of inflow streams into the crater lake of Rano Raraku strongly implies that the input of new freshwater mainly derives from meteoric water and possibly from sea spray. As Rapa

Nui is a small island in the middle of the Pacific Ocean, the main source of precipitation is evaporation and convective activity from the surrounding ocean. Precipitation that directly derives from a marine source often contains higher concentrations of salts than does precipitation from evaporation and convection from terrestrial freshwater sources. Our results are consistent with this idea, strongly indicating precipitation originated from evaporation of ocean water. The major element concentrations in filtered water samples (table S1) demonstrate that Cl and Na were particularly abundant in the dissolved fraction (mean concentrations of 968 ± 28 and 609 ± 12 mg L^{-1}, respectively). The Na/Cl mean ratio is 0.63, slightly higher – but comparable to – the reference Na/Cl ratio in seawater (0.56) (Millero, 1974). Major ions (Cl^-, SO_4^{2-}, Na^+, K^+, Ca^{2+} and Mg^{2+}) in Rano Raraku correlate (r^2 = 0.970; slope of 18 ± 1; *p*-value < 0.001) with typical seawater (Millero, 1974) providing further evidence of a direct marine source. The Na/Cl ratio in Rano Raraku water (0.63 ± 0.01) is also comparable to that reported in Hanga Roa meteoric waters collected in 2002-2003 (3-14 mg L^{-1},0.74 ± 0.12; *p*-value = 0.016; Herrera and Custodio, 2008). Therefore, Na^+ and Cl^- input almost completely results from meteoric waters, in agreement with the general scarcity of Na^+ found in soils in the Rano Raraku catchment (table S4). These values are within the range of the Eriksson (1952) equation that suggests Cl^- values between 6 and 12 mg L^{-1} (in meteoric waters at ~1 km from the sea).

The isotopic composition of Rano Raraku water ranges between 25 ± 2 ‰ and 4.0 ± 0.1 ‰ for δ^2H and $\delta^{18}O$ respectively. These positive values indicate that the lake has predominantly been affected by evaporation processes. A simplified version of the Craig and Gordon (1965) model that assumes no resistance to mixing in the liquid phase can model closed water system (such as a small lake), where no inflow or outflow is present and the isotopic composition is driven only by evaporation (Gonfiantini, 1986). The observed slope of the evaporation line (LEL) predicted by the Craig-Gordon model is:

$$S_{LEL} = \frac{\left[\frac{h(\delta_A - \delta_P) + (1+\delta_P)\left(\varepsilon_K + \frac{\varepsilon^+}{\alpha^+}\right)}{h - \varepsilon_K - \frac{\varepsilon^+}{\alpha^+}}\right]_{\delta D}}{\left[\frac{h(\delta_A - \delta_P) + (1+\delta_P)\left(\varepsilon_K + \frac{\varepsilon^+}{\alpha^+}\right)}{h - \varepsilon_K - \frac{\varepsilon^+}{\alpha^+}}\right]_{\delta^{18}O}} \quad (1)$$

where δ_A is the isotopic composition of atmospheric moisture, δ_P is the isotopic composition of precipitation, h is the relative humidity, α^+ is the liquid-vapor equilibrium isotopic fractionation, ε^+ is the equilibrium isotopic separation between liquid and vapor, and ε_K is the equivalent kinetic isotopic separation based on tunnel experiments (Gonfiantini, 1986; Horita and Wesolowski, 1994; Gat, 1996; Gibson et al., 2008; Herrera and Custodio, 2008). The α^+ value is solely a function of temperature that, in this study, was assumed to be equal to 20 °C. This temperature is based on the fact that Rapa Nui temperatures do not change significantly during the year, as they only range between 18 °C in July/September and 23 °C in January/March (Herrera and Custodio, 2008). Relative humidity was set to h = 0.79 (Schaller et al., 1997). Based on these assumptions, LEL was 6.02, slightly lower than the mean meteoric water line ($\delta^2H = 8\cdot\delta^{18}O + 10‰$; blue line in fig. 2). Forcing the straight line through the δ^2H and $\delta^{18}O$ mean precipitation values, we obtained the equation $\delta^2H = 6.02\cdot\delta^{18}O + 4.04‰$ (red line in fig. 2). The isotopic composition of the Rano Raraku water matches the model, as the points (in red) lie close to the red line.

Figure 2. Isotopic composition of rainfall on Rapa Nui (black dots; from Herrera and Custodio, 2008) and Rano Raraku water in 2017 (red dots). Mean meteoric water line (blue), rainfall best fit (black line) and equation straight line derived from the evaporation model (red line).

3.2 Chloride mass balance

In order to evaluate the precipitation-evaporation balance during the last few decades, we estimate the chloride mass balance. We assume that Cl^- input predominantly derives from saline rainfall, while evaporation further increases the salinity of the lake water. In 2017, which was the driest year since 1967 (Center for Climate and Resilience Research, http://www.cr2.cl/datos-de-precipitacion/?cp_Precipitacion=1), the mean Cl^- concentration in Rano Raraku lake water was 968 mg L^{-1}. Considering a mean lake depth of 0.6 m and assuming a lake surface area of 0.0706 km^2, the total mass of chloride in the lake was ~ 41,000 kg. As mentioned previously, the rainfall regime differs across Rapa Nui, where precipitation is more abundant in higher elevations including Mount Terevaka (507 masl) than in the rest of the island (Herrera and Custodio, 2008). The only multi-decadal (1954-2018) precipitation record for Rapa Nui is from the Mataveri airport gauge in Hanga Roa, (fig. 3). Although not representative of the overall precipitation regimes in the island, this site is comparable to Rano Raraku in terms of annual rainfall (Puleston et al., 2017). Therefore, mean annual precipitation at Rano Raraku can be estimated around 1,200 ± 100 mm yr^{-1} (Puleston et al., 2017) with a Cl^- content of ~ 8 mg L^{-1}.

Under these assumptions, we estimate that the amount of Cl^- input to the basin, provided solely by meteoric water, averaged 680 kg y^{-1} (at 2017 lake level conditions). The precipitation record was used to calculate the amount of Cl^- provided solely by precipitation since 1954 and resulted in a total of approximately 40,000 kg. This value is surprisingly close to the 41,000 kg of Cl^- estimated in Rano Raraku in 2017. In 1990, the electrical conductivity of the lake water was 640 µS cm^{-1} (i.e. 0.64 dS m^{-1}; Geller, 1992). Using the equation proposed by Peinado-Guevara et al. (2012) ($[Cl^-]$ (meq L^{-1}) = 4.928 EC (dS m^{-1})) we can estimate a concentration of 112 mg L^{-1}. Considering a mean lake depth of ~ 3 m (Geller, 1992) and a surface area of 0.09 km^2 (Dumont et al., 1998) in 1990, we can estimate that 30,000 kg of Cl^- were present in the lake at that moment. As the lake depth ranged between ~ 0.5

to ~ 1.5 m during the 2017 drilling campaign, this estimate is likely at the high extreme. In this study the precipitation record was also used to determine the amount of Cl^- supplied by rainfall. Assuming the chloride content to be constant in rainfall (8 mg L^{-1}), the amount of the ion that accumulated in the lake since 1954 is ~ 29,000 kg. This value is quite close to the amount of chloride estimated in 1990 (i.e. 30,000 kg). This data from 1990 and 2017 suggest that Cl^- only started to accumulate in Rano Raraku lake since the 1950s, even though the lake formed thousands of years ago (> 50,000 cal yrs BP). This accumulation could be due to the fact that Rano Raraku lake is a naturally closed basin and that the insertion of a pipe in 1958 (Flenley and Bahn, 2003) could have artificially drained out much of the water that was present in the 1950-60s.

Although this calculation requires several assumptions, these results suggest that (a) the source of water in Rano Raraku is solely meteoric and (b) Rano Raraku is a closed system where the lake level is controlled only by evaporation-precipitation balance. The isotopic composition of the water in 2017 agrees with the hydrological closed system model where the isotopic composition is affected solely by evaporation. During the last 30 years, the lake level was reported to remain between the depths of 2 and 4 m (Geller, 1992; Dumont et al., 1998; Saez et al., 2009; Cañellas-Boltà et al. 2012; Horrocks et al. 2012), although current lake levels are less than 2 m. These fluctuations follow the same general trend as the precipitation record (fig. 3), further suggesting that the evaporation-precipitation balance is the only natural lake level control.

Figure 3. Monthly precipitation at Mataveri, Rapa Nui (Easter Island) (Center for Climate and Resilience Research, http://www.cr2.cl/datos-de-precipitacion/?cp_Precipitacion=1).

3.3 Redox conditions

In September 2017, Rano Raraku water levels were low (< 2 m) and higher concentrations of NH_4^+ than nitrates in water, indicating a potential lack of nitrification reactions indicate highly anoxic conditions. In oxygenated waters, ammonium is subject to nitrification, transforming NH_4^+ into N-oxidized forms, such as NO_3^- (Quiròs, 2003). At relatively low concentrations of dissolved oxygen, nitrification reactions of NH_4^+ cease and, consequently ammonium concentrations increase (Bahteria and Jain, 2016). Besides the degradation of autochthonous organic matter, additional sources of NH_4^+ include fertilizers and animal feces. In recent times, the fecal input from free-roaming horses that often graze inside the crater may be a possible NH_4^+ source. Horses, as well as sheep and cattle, were introduced by the missionaries who settled in 1864 (Flenley and Bahn, 2003). However, although sheep were numerous for almost a century after 1870, they were then largely replaced by cattle and horses.

The higher NH_4^+ concentrations likely contribute to the slightly alkaline pH of 7.8 measured during our sampling campaign, which differs from the slightly acidic conditions (pH of 6.3) observed in 1990 by Geller (1992). At Rano Raraku, the total organic carbon content of the sediment samples was 18.3 % and 16.9 % in WSI and bed sediments, respectively. Organic decomposition can occur in both aerobic and anaerobic conditions, with a complex mechanism that involves several steps, favored by the presence of electron acceptors such as nitrates, sulphates and Fe^{3+}. In water samples sulfates were significantly depleted (i.e. SO_4^{2-}/Cl^- ratio ~ 10^{-3}) compared to seawater (SO_4^{2-}/Cl^- = 0.14) suggesting that sulfate reduction may be involved in the decomposition of the organic matter.

In the aqueous phase, mean Mn and Fe concentrations were 4 ± 1 and 2.0 ± 0.5 µg L^{-1}, respectively, resulting in a relative enrichment of Mn (Mn/Fe = 2.0 ± 0.9). In the SPM, the

concentrations of the redox-sensitive elements Mn and Fe were 58 ± 18 and 868 ± 515 µg L^{-1}, respectively (table S2). This large concentration range is related to the variability in SPM mass (data were normalized by the volume of water). The Mn/Fe was 0.07 ± 0.02 (with a variability less than 30 %). The Fe and Mn concentrations were 96 ± 14 mg g^{-1} and 2.3 ± 0.4 mg g^{-1} in WSI (table S3 and fig. 4). In bed sediments, Fe slightly increases while Mn substantially decreases. Consequently, the Mn/Fe ratio is higher in WSI (0.024 ± 0.003) than in bed sediments (0.015 ± 0.002). In the topsoil samples (table S4 and fig. 4), Fe is particularly abundant in the form of oxyhydroxide minerals, as also observed by Horrocks et al. (2012). However, the topsoil Mn/Fe ratio is 0.020 ± 0.005, close to the Mn/Fe in the upper crust (i.e. 0.020; Rudnick and Gao, 2014) and in volcanic glass in Rano Raraku hyalotuff (i.e. Mn/Fe ~ 0.025 ± 0.007; Gioncada et al. 2010). The Mn/Fe ratios are substantially lower than values found both in Rano Raraku water and in SPM (t-test of the means provides *p*-value lower than 10^{-4}).

The cycling of Mn and Fe in lake sediments is associated with the oxic/anoxic conditions of the water body (Abesser et al., 2010) and is often used as a proxy in paleoclimate reconstructions (Naeher et al., 2013; Vegas-Vilarrúbia et al., 2018; Żarczyński et al., 2019). External sources of Mn and Fe include catchment erosion and redox-related dissolution of minerals. Internal biochemical and/or physical processes are responsible for the increasing/decreasing concentrations of these elements in the water column, as a result of remobilization and redox-controlled release from the sediment (Murray, 1987; Balistrieri et al. 1992; Giles et al., 2016). In Rano Raraku, the external input of Mn and Fe derives from catchment erosion or wind transport from the surrounding areas, where the Mn/Fe ratio in topsoil is 0.020 ± 0.005 on average, ranging from 0.017 to 0.029. Fig. 5 presents a schematic explanation of the cycling of Mn and Fe at Rano Raraku. The reducing (anoxic) environment favors the reduction of Mn(IV) and Fe(III) in the sediment that diffuse as Mn(II) and

Fe(II) through the water body if it is anoxic. Due to the higher mobility of Mn(II) ions, they diffuse faster than Fe(II), enriching the water-sediment interface with Mn (Mn/Fe = 0.024 ± 0.003), compared to the underlying sediment (Mn/Fe = 0.015 ± 0.002). In the water, Mn(II) is stabilized by the reducing conditions and prevails in dissolved forms (Mn/Fe = 2.0 ± 0.9). The re-oxidation of Fe is faster than Mn, promoting the formation of iron oxy-hydroxides that adsorb onto the SPM, where the SPM is then enriched in Fe compared to the dissolved fraction (Mn/Fe = 0.07 ± 0.02), but more depleted than the sediment. This mechanism implies that under anoxic conditions, Mn/Fe is expected to increase in the water body, while decreasing in the sediment, as supported by concentration data (fig. 5).

In addition to the Mn/Fe ratio, Cr and V can also reconstruct redox conditions in lacustrine environments. Under anoxic and more reducing conditions, vanadyl (VO^{2+}) and Cr(III) ions interact differently with surface particles and organic ligands. VO^{2+} prevails in dissolved forms, thus enriching the Cr/V ratio in sediments. In the aqueous phase Cr and V concentrations were 61 ± 14 and 116 ± 39 ng L^{-1} respectively, providing Cr/V values of 0.6±0.2. In the SPM, Cr and V concentrations were 6.4 ± 0.7 and 2.2 ± 0.4 µg L^{-1}, resulting in a Cr/V ratio of 3.0 ± 0.2. The Cr/V ratio for SPM was significantly higher (p-value < 10^{-4}) than the dissolved fraction ratio. Cr/V ratios in WSI and bed sediments do not show substantial differences, although these values (Cr/V = 0.039 ± 0.006 and 0.038 ± 0.006) are considerably lower than Cr/V in all of the other environmental compartments, indicating a relative depletion of Cr with respect to V. Vanadium content in topsoil samples was 155 ± 62 µg g^{-1} and, despite the high variability, does not significantly differ on average from the upper crustal concentration (p-value = 0.07; V = 97 µg g^{-1} from Rudnick and Gao, 2014). Conversely, the Cr concentration in soils is 64 ± 18 µg g^{-1}, which is significantly depleted when compared to the upper crust composition (Cr = 92 µg g^{-1} from Rudnick and Gao, 2014; *p*-value =

0.012). Therefore, the Cr/V value of 0.4 ± 0.1 in the soil is significantly lower than SPM but comparable to values found in water (p-value = 0.16).

Although Cr/V has lower values in the water (0.6 ± 0.2) and higher values in SPM (3.0 ± 0.2) the Cr/V ratio is very low in the water-sediment interface and in the underlying sediment. Since V is expected to form more soluble compounds in reducing environments, this occurrence in the sediment is unexpected. Vanadium equilibrium is sensitive to pH and the speciation of V under reducing conditions is still under debate (Gustafsson, 2019). In reducing sediments, a mixture of vanadyl (IV) and unknown vanadium (III) species is strongly complexed by organic compounds, while under oxic conditions vanadate (V) preferentially interacts with Fe (hydro)oxides. The Rano Raraku lacustrine system is particularly enriched both in Fe and in organic matter, and the pH increases with the eutrophication of the lake. Therefore, V may be entrapped in sediment, which is compatible with Cr/V values of 0.039 ± 0.006 in WSI and 0.038 ± 0.006 in the sediment underneath, meaning that using the Cr/V ratio as a proxy for redox conditions in Rano Raraku must be carefully evaluated.

Figure 4. Mean Mn/Fe and Cr/V ratios with standard deviations of the analyzed matrices: water, SPM (suspended particulate matter), WSI (water-sediment interface), BS (bed sediment), topsoil.

Figure 5. Scheme of the fractionation process of Mn and Fe in Rano Raraku.

3.4 Salinity and alkaline earth element fractionation

The Rano Raraku topsoil is generally depleted in alkaline earths and alkali metals especially Na and Ca. The Ca/Sr and Mg/Ca ratios are 47 ± 15 and 8 ± 4 (table S4), respectively. Although slightly higher, than the ratios from SPM and water, the soil ratios are within the same order of magnitude.

The Rano Raraku water is enriched in Mg and Ca (mean values of 53 ± 1 and 15 ± 1 mg L^{-1}), followed by Sr (mean value 622 ± 136 µg L^{-1}). The Ca/Sr and Mg/Ca ratios are 24 and 3.6, respectively. Magnesium and Ca are strongly partitioned in the SPM (143 and 59 µg L^{-1}) compared to the more soluble alkaline ions (Na and K), which were lower than 20 µg L^{-1} (table S2). The SPM differs from the dissolved fraction and is depleted in Sr (2 ± 1 µg L^{-1}), but the Ca/Sr and Mg/Ca ratios (35 ± 6 and 2.5 ± 0.4) in SPM do not markedly differ from water. In the WSI, which is defined as the uppermost 2 cm of the core, alkaline earths are significantly higher (Ca 151 ± 23 µg g^{-1}; Mg 2.3 ± 0.3 mg g^{-1}) than in the deeper section (between 3 and 5 cm from the sediment-water surface, Ca 47 ± 7 mg g^{-1}; Mg 1.7 ± 0.3 mg g^{-1}), as well as Ca/Sr (WSI: 5.4 ± 0.8; bed sediments: 3.5 ± 0.5). However, Mg/Ca is higher in bed sediments (36 ± 5) than WSI (15 ± 2) (table S3).

The precipitation of aragonite and calcite infers oscillations in water salinity in marine and lacustrine systems (Kelts and Hsü, 1978; Tesoriero and Pankow, 1996; Ito, 2002; Dissard et al., 2010). In more saline waters, high-Mg and high-Sr calcite or aragonite preferentially precipitate. The log-log plot of Mg/Ca *vs* Ca/Sr is in agreement with this process ($r^2 = 0.983$; p-value = 0.0089; fig. 6). Except in the topsoil, the increase in Mg/Ca from SPM to bed sediments corresponds with a decrease in Ca/Sr. Therefore, the precipitation of carbonates enriched in Mg and Sr depletes the water column of these elements, while the WSI is more depleted than bed sediments, likely due to the mixing effect exerted by water. However, the topsoil is not directly involved in this fractionation process (fig. 6).

As a first approximation, we assume that Mg/Ca (or Ca/Sr) responds linearly to a change in salinity, as observed in previous studies (Dissard et al., 2010)

Figure 6. Log-log plot of Mg/Ca vs Ca/Sr values in different environmental components. Topsoil (red point) does not lie on the line because it is not involved in the fractionation process occurring in the lake.

Figure 7. Ce/Ce* and LREE/HREE mean ratios with standard deviation in the analyzed matrices: water, SPM (suspended particulate matter), WSI (water-sediment interface), BS (bed sediment), topsoil.

3.5 REEs fractionation and lake level oscillations

Average concentrations of REEs in Rano Raraku water range from 0.3 to 3.7 ng L^{-1}, where Ce and Nd are the most abundant. In this paper, we propose a diagnostic ratio between LREE (La, Pr and Nd) and HREE (Er, Tm, Yb and Lu) to investigate possible fractionation processes. The ratio LREE/HREE is defined as the following:

$$\left(\frac{LREE}{HREE}\right)_i = \frac{4\cdot\left(\frac{[La]_i}{[La]_c}+\frac{[Pr]_i}{[Pr]_c}+\frac{[Nd]_i}{[Nd]_c}\right)}{3\cdot\left(\frac{[Er]_i}{[Er]_c}+\frac{[Tm]_i}{[Tm]_c}+\frac{[Yb]_i}{[Yb]_c}+\frac{[Lu]_i}{[Lu]_c}\right)} \qquad (2)$$

where the subscript *i* refers to the concentration in samples and the subscript *c* refers to the upper crust concentration (Rudnick and Gao, 2014). As shown in table S1 and fig. 7, the Rano Raraku water has LREE/HREE = 0.10 ± 0.02, indicating a depletion (enrichment) in LREE (HREE). Among the REEs, Ce is of particular interest as its concentration is affected by a combination of environmental factors and processes such as leaching, runoff and redox equilibria. This combination is mainly due to the higher solubility of reduced Ce(III) compared to oxidized Ce(IV) species, which differs from the other REEs that exhibit only one redox state (III) in the environment (except Eu). The cerium anomaly (Ce/Ce*) is defined as the following (Elderfield et al, 1982):

$$\frac{Ce}{Ce^{*}} = \frac{3\cdot\left(\frac{[Ce]_i}{[Ce]_c}\right)}{2\cdot\left(\frac{[La]_i}{[La]_c}\right)+\left(\frac{[Nd]_i}{[Nd]_c}\right)} \qquad (3)$$

We also use equation (2) to calculate Ce/Ce* in all of the investigated matrices. In water, Ce/Ce* was 0.6 ± 0.1, suggesting a depletion in Ce with reference to the value expected when no fractionation occurs (i.e. Ce/Ce* = 1).

In the Rano Raraku SPM, REE concentrations range between a few to hundreds of ng L^{-1} where, similarly to the surrounding water, Ce and Nd are the most abundant REEs. The LREE/HREE ratio is 0.76 ± 0.03, which is significantly higher than in the dissolved phase (p-value < 10^{-4}), indicating a REE fractionation with an enrichment (depletion) of less (more) soluble LREEs (HREEs) in SPM. Similarly, Ce/Ce*(1.02 ± 0.04) is significantly higher in SPM than in water (p-value < 10^{-4}).

In sediments, REEs range between tens of ng to a few µg per gram, with higher concentrations of Ce, Nd and La than the other REEs. The LREE/HREE ratio is 0.42 ± 0.06 and 0.28 ± 0.04 in WSI and bed sediments, respectively. The Ce anomaly slightly exceeds unity in both WSI (1.3 ± 0.2) and bed sediments (1.5 ± 0.2), indicating an enrichment in Ce, which is similar the Ce anomaly in SPM.

Rare earth elements in topsoil samples contain concentrations between 1 and 150 µg g^{-1} and are particularly enriched in Ce and to a lesser extent in Nd and La. The LREE/HREE ratio in soils was 0.4 ± 0.1, significantly higher than in the water, but lower than in SPM and comparable to ratios in the sediment. The cerium anomaly is more variable than in the other matrices with values (Ce/Ce* = 4 ± 2) that are substantially higher than in both water and SPM, indicating a general excess of Ce in topsoil.

The REE fractionation is a complex process that can be due to leaching during runoff, or the formation of soluble/insoluble compounds in the aquatic environment when organic or inorganic ligands are present. Assuming that the marine source contribution to REEs is negligible due to the low concentration of REEs in the Pacific Ocean (De Baar et al., 1985), the comparison between LREE/HREE in the different compartments suggests that REEs fractionate inside the water body. This fractionation is driven by the difference in solubility between LREE and HREE in aqueous solutions, where HREEs are more soluble than LREEs (Nelson et al., 2003). In Rano Raraku, a possible mechanism may involve the preferential LREE adsorption onto the particulate fraction bonded to Fe-Mn oxides or organic material, or an enhanced solubility of HREEs due to the presence of organic anions, hydroxides and carbonates. Although Cl^- can increase LREE solubility (Tsay et al., 2014), this mechanism does not seem to dominate in Rano Raraku despite the high Cl^- concentration, in fact LREE concentrations remain relatively low in water with respect to the other matrices.

Cerium can provide additional information on fractionation processes. In the water body, Ce/Ce* shows a slight depletion in the dissolved phase and a moderate enrichment in SPM and sediments. The reducing environment stabilized the relatively more soluble Ce(III) complexes, thus limiting the fractionation inside the water body. The slightly higher values in the underlying sediment, although not remarkable, may be ascribed to conditions in the recent past when the lake was slightly more oxic. Therefore, although Ce/Ce* fractionation resembles the LREE/HREE partitioning, the Ce anomaly may be potentially more sensitive to redox conditions than LREE/HREE, where the latter is more sensitive to the chemical composition of the water column. Therefore, REE analysis suggests that these elements are more sensitive to the dynamics occurring in the water body rather than to potential changes in the transport of exogenous material. Airborne transport has a potential role in terrigenous material input, as field observations confirm the presence of moderately intense dusty

winds. Erosion or leaching processes during lake level fluctuations were likely limited during the last decades. However, all of these factors may have been more influential in the past, although their interpretation may be affected by contrasting factors. For example, if leaching increases during lake level rise, a Ce/Ce* depletion should be observed in the lake system, as Ce is less soluble than other REEs. Nevertheless, the lake level rise is accompanied by more oxic conditions that favor the Ce(IV) precipitation in the sediment. Therefore, the variation in Ce/Ce* in the sediment depends on the balance between these factors. In topsoil samples, a LREE/HREE and Ce/Ce* significantly correlate when using a log-log scale ($r^2 = -0.905$; p-value = 0.013). LREE/HREE increases with increasing the elevation, while Ce/Ce* decreases (fig. 8). This trend is consistent with past lake level oscillations. At relatively higher elevations, the topsoil may have been directly exposed to lake water only during times of exceptionally high lake levels, while at lower elevations, the soil was in contact with water for a longer time. Lake water preferentially solubilizes HREE rather than LREE, and REE rather than Ce. Since these solubilization processes are kinetically controlled, the longer that soil was exposed to water, the lower the LREE/HREE and the higher the Ce/Ce*.

Figure 8. LREE/HREE and Ce anomaly vs elevation of topsoil samples from the Rano Raraku internal crater.

3.6 Anthropological implications

Chloride mass balance demonstrated that without any natural water removal, the salinity of the lake is driven solely by evaporation-precipitation balance and chlorides are constantly introduced in

the lacustrine system by saline rainfalls. The modern concentration of ~ 1 g L^{-1} is a consequence of a combination of evaporation and precipitation that was reset when the lake was artificially drained out during the 50s. Yet, assuming that no natural drainage has ever occurred, what could be the salinity of Rano Raraku water when this site was the quarry during the Ancient Cult period? The oldest sediment is dated back to ~34,000 years ago (Canellas-Boltà et al. 2016) and without any natural water removal, assuming an input of 660 kg y^{-1} and a lake level optimistically high ~20 m, between 1000 and 1500 AD, the salinity of the water should have been of about 8.6 - 8.8 g L^{-1}. Human tolerance towards chloride in drinking water is theoretically equal to the salinity of blood plasma, that is ~ 9 g L^{-1} (Crawford and Harris 2011). Nevertheless, the habitual consumption of water with a salinity of 1.7 g L^{-1} in southern Israel revealed the occurrence of several health disorders (Berlyne and Morag 1972), while the consumption of freshwater containing chlorides in a concentration < 250 mg L^{-1} is recommended, in order to not to detect a salty taste (WHO, 2003). Norton (1992) noted that Easter Island is the only major Polynesian island where drinking brackish water was a common occurrence. Therefore, although the presence of human activity at Rano Raraku during the apogee of the Ancient Cult may have been favored by the presence of freshwater, the potential high salinity of the water places several questions about its use. The absence of historical European accounts mentioning that the lake water was directly used as a source of freshwater for drinking during prehistory and the lack of evidence of residential occupation nearby (Brosnan et al. 2018) are in agreement with our considerations. Rano Raraku was affected by severe droughts between 1570 and 1720 AD (Canellas-Boltà et al. 2013; Rull et al. 2016b), and this may had promoted a significant cultural shift from the Ancient to the Birdman cult (Edwards and Edwards, 2013). Rano Raraku droughts coincided with the move of the cultural centre to Rano Kau, although the lack of its freshwater availability was likely not a direct driving factor. Rather, lake Raraku severe and

prolonged droughts reflect a significant reduction of freshwater availability all along the island. Undoubtedly, freshwater availability in Easter Island was - and it is still - a considerable limitation. Archaeological evidence suggested that freshwater management has been a timeless issue. Historical accounts reported that ancient Rapa Nui people used groundwater for drinking (Dos Passos 1971; Forster and Kahn 1968; Richards 2008) and nowadays (2017 AD) we observed horses drinking water along the coastline from freshwater seepage. To overcome this issue, ancient Rapa Nui people built structures similar to wells (*puna*) that were used to trap coastal groundwater (Englert, 1948; Metraux, 1940) as well as shallow artificial basins carved into basalt rocks (*taheta*) used to hold rainwater (Metraux, 1957). More recently, Di Napoli et al. 2019 found a significant correlation between the location of monumental platforms (*ahu*) and the presence of freshwater sources. Thus, rather than a permanent freshwater lagoon, groundwater was likely a more important source of drinking water for ancient Rapa Nui people. Nevertheless, discharged coastal groundwater is brackish. The salinity of groundwater in Easter Island nowadays ranges from ~80 mg L^{-1} in Vaitea up to ~1.9 g L^{-1} along the coast (Herrera and Custodio 2008). Besides brackish, the available water sources in Easter Island are limited and cannot sustain the water demand of the present population. In the past, coincidentally with prolonged droughts occurred at Rano Raraku, even groundwater availability was significantly reduced all along the island, potentially contributing to trigger the social instability in a densely populated island of estimated 10-20 thousands of people (Puleston et al. 2017; Fisher, 2005; Fagan, 2008), although these demographic estimates have been questioned by several authors (Lipo et al. 2018).

3. Conclusions

Studies of current geochemical characteristics help interpret past environmental changes. Rapa Nui (Easter Island) is often used as an example of a location where human influence on environmental change is closely linked and amplified due to Rapa Nui's size and isolation. However, few studies on Rapa Nui provide modern baselines against which these past changes can be compared. The interactions between soil, water, and lake sediments from Rano Raraku demonstrate a modern system that has substantially changed after the introduction of an outlet pipe in the 1950s, and then the return to a closed basin. The stable isotopic ratios (25 ± 2 ‰ and 4.0 ± 0.1 ‰ for δ^2H and δ^{18}O respectively) and the Craig-Gordon model, demonstrate that the origin of Rano Raraku water is solely meteoric, originating from the nearby Pacific Ocean as highlighted by dissolved ions. The high salinity of Rano Raraku waters, due to the influence of coastal rainfall, affects the precipitation balance of alkaline earths. The topsoil is essentially depleted in alkaline earths yet the uppermost 2 cm of the core, contain relatively high alkaline earth concentrations (Ca 150 ± 20 µg g^{-1}; Mg 2.3 ± 0.3 mg g^{-1}) that are double those from the deeper section (between 3 and 5 cm from the sediment-water surface, Ca 50 ± 7 mg g^{-1}; Mg 1.7 ± 0.2 mg g^{-1}). The lake level is mainly dependent on the evaporation-precipitation balance, as Rano Raraku is essentially a closed system with scarce influence from runoff where (between 1950 until the last years of 1970s) the insertion of a pipe provided the only outflow. Rano Raraku is currently anoxic, as demonstrated by higher ammonium than nitrate concentrations. Horses that graze on the shores of the lake may be responsible for the increasing ammonium concentrations since the 1990s, as well as the increase in lake pH since the 1990s.

Field observations and the balance of redox-sensitive elements such as Mn, Fe, Cr and V are consistent with dominant reducing conditions in the basin, likely related to a current eutrophic state further supported by the high sedimentary organic carbon values. Ratios of Ce/Ce* are slightly depleted in the lake water yet are enriched in both SPM and sediments. The current reducing

conditions stabilize the more soluble Ce(III) complexes. However, in the recent past, the lake was more oxic, resulting in slightly higher Ce/Ce* values in the underlying sediment, LREE/HREE values in the water and sediment agree with this change from past oxic to current reducing conditions, even though LREE/HREE is more sensitive to the chemical composition of the water column. The sensitivity of the lake to this human interference is important to consider when investigating past changes determined from Rano Raraku lake sediments as the Rano Raraku surrounding area was and has been an important cultural site and quarry for hundreds of years.

In addition to these conclusions, one of the most important aspects of this study is the connection between Rano Raraku and the decline of the Ancient Cult. In fact, this study highlights the enduring difficulties of the ancient Rapa Nui people to live in this island were, even today, the supply of freshwater is challenging.

Acknowledgments

This work was supported by Ca' Foscari University of Venice through the Progetto di Ateneo 2015. We thank the Corporación National Forestal and, in particular, Lilian Gonzalez Nualart, Ninoska Cuadros and Michel Pate. We also thank the Consejo de Monumentos Nacionales and, in particular, Maria Gabriela Atallah Leiva and Merahi Atam Lopez. Elga (High Wycombe, UK) is acknowledged for providing the Purelab Ultra system employed for producing the ultrapure water (18 MΩ cm^{-1} resistivity) used throughout the study. Field and laboratory support was provided by CSDCO/LacCore, University of Minnesota, funded by the US National Science Foundation. DM was partially supported by NSF BCS-1832486. Any use of trade, firm, or product names is for descriptive purposes only and does not imply endorsement by the U.S. Government. All the data collected for this research are available on Mendeley Data using the following link https://data.mendeley.com/datasets/k93rp3p4pd/1.

References

Abesser, C., Robinson, R., and Robinson, R. (2010). Mobilisation of iron and manganese from sediments of a Scottish Upland reservoir. Journal of Limnology, 69(1), 42–53. https://doi.org/10.4081/jlimnol.2010.42

Aide, M. T., and Aide, C. (2012). Rare Earth Elements: Their Importance in Understanding Soil Genesis. ISRN Soil Science, 2012, 783876. https://doi.org/10.5402/2012/783876

Azizi, G., and Flenley, J. (2008). The last glacial maximum climatic conditions on Easter Island. Quaternary International, 184(1), 166–176. https://doi.org/10.1016/j.quaint.2007.09.027

Baker, P. E., Buckley, F., and Holland, J. G. (1974). Petrology and geochemistry of Easter Island. Contributions to Mineralogy and Petrology, 44(2), 85–100. https://doi.org/10.1007/BF00385783

Balistrieri, L. S., Murray, J. W., and Paul, B. (1992). The cycling of iron and manganese in the water column of Lake Sammamish, Washington. Limnology and Oceanography, 37(3), 510–528. https://doi.org/10.4319/lo.1992.37.3.0510

Barker, P. A., Roberts, N., Lamb, H. F., van der Kaars, S., and Benkaddour, A. (1994). Interpretation of Holocene lake-level change from diatom assemblages in Lake Sidi Ali, Middle Atlas, Morocco. Journal of Paleolimnology, 12(3), 223–234. https://doi.org/10.1007/BF00678022

Battistel, D., Roman, M., Marchetti, A., Kehrwald, N. M., Radaelli, M., Balliana, E., Toscano, G., and Barbante, C. (2018). Anthropogenic impact in the Maya Lowlands of Petén, Guatemala, during the last 5500 years. Journal of Quaternary Science, 33(2), 166–176. https://doi.org/10.1002/jqs.3013

Berlyne, G. M., and Morag, M. (1972). Metabolic effects of drinking brackish water. *Desalination*, *10*(2), 215-219.

Bhateria, R., and Jain, D. (2016). Water quality assessment of lake water: A review. Sustainable Water Resources Management, 2(2), 161–173. https://doi.org/10.1007/s40899-015-0014-7

Brosnan, T., Becker, M.W., Lipo, C.P. 2018 Coastal groundwater discharge and the ancient inhabitants of Rapa Nui (Easter Island) Chile. Hydrogeol J. https://doi.org/10.1007/s10040-018-1870-7

Cañellas-Boltà, N., Rull, V., Sáez, A., Margalef, O., Giralt, S., Pueyo, J. J., Birks, H. H., Birks, H. J. B., and Pla-Rabes, S. (2012). Macrofossils in Raraku Lake (Easter Island) integrated with sedimentary and geochemical records: Towards a palaeoecological synthesis for the last 34,000 years. Quaternary Science Reviews, 34, 113–126. https://doi.org/10.1016/j.quascirev.2011.12.013

Cañellas-Boltà, Núria, Rull, V., Sáez, A., Margalef, O., Bao, R., Pla-Rabes, S., Blaauw, M., Valero-Garcés, B., and Giralt, S. (2013). Vegetation changes and human settlement of Easter Island during the last millennia: A multiproxy study of the Lake Raraku sediments. Quaternary Science Reviews, 72, 36–48. https://doi.org/10.1016/j.quascirev.2013.04.004

Cañellas-Boltà, Núria, Rull, V., Sáez, A., Margalef, O., Pla-Rabes, S., Valero-Garcés, B., and Giralt, S. (2016). Vegetation dynamics at Raraku Lake catchment (Easter Island) during the past

34,000years. Palaeogeography, Palaeoclimatology, Palaeoecology, 446, 55–69. https://doi.org/10.1016/j.palaeo.2016.01.019

Craig, H., Gordon, L. I., and Conference on Stable Isotopes in Oceanographic Studies and Paleotemperatures (Eds.). (1965). Deuterium and oxygen 18 variations in the ocean and the marine atmosphere. Consiglio nazionale delle richerche, Laboratorio de geologia nucleare.

Crawford A, Harris H. I.V. fluids What nurses need to know. Nursing. 2011 May;41(5):30-8; quiz 38-9. doi: 10.1097/01.NURSE.0000397458.92532.44.

Davison, W., Woof, C., and Rigg, E. (1982). The dynamics of iron and manganese in a seasonally anoxic lake; direct measurement of fluxes using sediment traps. Limnology and Oceanography, 27(6), 987–1003. https://doi.org/10.4319/lo.1982.27.6.0987

Davison, William. (1993). Iron and manganese in lakes. Earth-Science Reviews, 34(2), 119–163. https://doi.org/10.1016/0012-8252(93)90029-7

De Baar, H. J. W., Bacon, M. P., Brewer, P. G., and Bruland, K. W. (1985). Rare earth elements in the Pacific and Atlantic Oceans. Geochimica et Cosmochimica Acta, 49(9), 1943–1959. https://doi.org/10.1016/0016-7037(85)90089-4

Dean, J. R., Eastwood, W. J., Roberts, N., Jones, M. D., Yiğitbaşıoğlu, H., Allcock, S. L., Woodbridge, J., Metcalfe, S. E., and Leng, M. J. (2015). Tracking the hydro-climatic signal from lake to sediment: A field study from central Turkey. Journal of Hydrology, 529, 608–621. https://doi.org/10.1016/j.jhydrol.2014.11.004

Diamond, J. (2005). Collapse: How societies choose to fail or succeed. Penguin.

Di Napoli, R.J., Lipo, C.P., Brosnan, T., Hunt, T.L. Hixon, S., Morrison A.E., Becker, M. 2018. Rapa Nui (Easter Island) monument (ahu) locations explained by freshwater sources. PLOS ONE. 14, e0210409. https://doi.org/10.1371/journal.pone.0210409

Dissard, D., Nehrke, G., Reichart, G. J., and Bijma, J. (2010). The impact of salinity on the Mg/Ca and Sr/Ca ratio in the benthic foraminifera Ammonia tepida: Results from culture experiments. Geochimica et Cosmochimica Acta, 74(3), 928–940. https://doi.org/10.1016/j.gca.2009.10.040

Dos Passos J (1971) Easter Island: island of enigmas. Doubleday, Garden City, NY, 216 pp

Dumont, H. J., Cocquyt, C., Fontugne, M., Arnold, M., Reyss, J.-L., Bloemendal, J., Oldfield, F., Steenbergen, C. L., Korthals, H. J., and Zeeb, B. A. (1998). The end of moai quarrying and its effect on Lake Rano Raraku, Easter Island. Journal of Paleolimnology, 20(4), 409–422.

Edwards, E., Edwards, A. 2013. When the universe was an island. Exploring the cultural and spiritual cosmos of the ancient Rapa Nui. Hangaroa press, Hangaroa.

Elderfield, H., and Greaves M.J., . (1982). The rare earth elemments in seawater. The Rare Earth Elements in Seawater.

Englert, S. 1948. La Tierra de Hoto Matu'a: Historia, etnologia, y lengua de Isla de Pascua. San Francisco.

Eriksson, E. (1952). Composition of atmospheric precipitation. Tellus, 4(4), 280–303.

Fagan, B. 2008. The great warming: climate change and the rise and fall of civilizations. New York, NY. Bloomsbury.

Fisher, S.R. 2005. Island at the end of the world: the turbulent history of Easter Island. London. Reaktion books.

Flenley, J., and Bahn, P. (2003). The enigmas of Easter Island: Island on the edge (2nd ed.). Oxford University Press.

Flenley, J. (1996). Further evidence of vegetational change on Easter Island. South Pacific Study, 16(2), 135–141.

Flenley, J. (1993). The palaeoecology of Easter Island, and its ecological disaster. Easter Island Studies. Oxbow Monograph, 32, 27–45.

Flenley, J., and King, S. M. (1984). Late quaternary pollen records from Easter Island. Nature, 307(5946), 47–50.

Forster G, Kahn RL (1968) Georg Forsters Werke [The works of George Forster]. Brandenburgische Akademie der Wissenschaften, Berlin, 278 pp.

Gat, J. R. (1996). Oxygen and hydrogen isotopes in the hydrologic cycle. Annual Review of Earth and Planetary Sciences, 24(1), 225–262.

Geller, W. (1992). The temperature stratification and related characteristics of Chilean lakes in midsummer. Aquatic Sciences, 54(1), 37–57.

Gibson, J. J., Birks, S. J., and Edwards, T. W. D. (2008). Global prediction of δA and $\delta^{2}H$- $\delta^{18}O$ evaporation slopes for lakes and soil water accounting for seasonality. Global Biogeochemical Cycles, 22(2).

Giles, C. D., Isles, P. D., Manley, T., Xu, Y., Druschel, G. K., and Schroth, A. W. (2016). The mobility of phosphorus, iron, and manganese through the sediment–water continuum of a shallow eutrophic freshwater lake under stratified and mixed water-column conditions. Biogeochemistry, 127(1), 15–34.

Gioncada, A., Gonzalez-Ferran, O., Lezzerini, M., Mazzuoli, R., Bisson, M., and Rapu, S. A. (2010). The volcanic rocks of Easter Island (Chile) and their use for the Moai sculptures. European Journal of Mineralogy, 22(6), 855–867.

Gonfiantini, R. (1986). Environmental isotopes in lake studies. Handbook of Environmental Isotope Geochemistry, 2, 113–168.

Gonzalez-Ferran, O., and Baker, P. E. (1974). Isla de Pascua. Field guide book, including the first easter island geological map 1: 50.000. IAVCEI International Symposium, Santiago, Chile, 32.

Gustafsson, J. P. (2019). Vanadium geochemistry in the biogeosphere–speciation, solid-solution interactions, and ecotoxicity. Applied Geochemistry, 102, 1–25.

Hamilton, S., Seager Thomas, M., and Whitehouse, R. (2011). Say it with stone: Constructing with stones on Easter Island. World Archaeology, 43(2), 167–190. https://doi.org/10.1080/00438243.2011.586273

Herrera, C., and Custodio, E. (2008). Conceptual hydrogeological model of volcanic Easter Island (Chile) after chemical and isotopic surveys. Hydrogeology Journal, 16(7), 1329–1348. https://doi.org/10.1007/s10040-008-0316-z

Holser, W. T. (1997). Evaluation of the application of rare-earth elements to paleoceanography. Palaeogeography, Palaeoclimatology, Palaeoecology, 132(1–4), 309–323.

Horita, J., and Wesolowski, D. J. (1994). Liquid-vapor fractionation of oxygen and hydrogen isotopes of water from the freezing to the critical temperature. Geochimica et Cosmochimica Acta, 58(16), 3425–3437.

Horrocks, M., Baisden, W. T., Flenley, J., Feek, D., Nualart, L. G., Haoa-Cardinali, S., and Gorman, T. E. (2012). Fossil plant remains at Rano Raraku, Easter Island's statue quarry: Evidence for past elevated lake level and ancient Polynesian agriculture. Journal of Paleolimnology, 48(4), 767–783. https://doi.org/10.1007/s10933-012-9643-0

Ito, E. (2002). Application of Stable Isotope Techniquesto Inorganic and Biogenic Carbonates. In Tracking environmental change using lake sediments (pp. 351–371). Springer.

Kelts, K., and Hsü, K. J. (1978). Freshwater carbonate sedimentation. In Lakes (pp. 295–323). Springer.

Leng, M. J., and Marshall, J. D. (2004). Palaeoclimate interpretation of stable isotope data from lake sediment archives. Quaternary Science Reviews, 23(7–8), 811–831.

Lipo, C., Hunt, T., Horneman, R., and Bonhomme, V. (2016). Weapons of war? Rapa Nui mata'a morphometric analyses (Vol. 90). https://doi.org/10.15184/aqy.2015.189

Lipo et al 2018 Commentary: Rain, Sun, Soil, and Sweat: A Consideration of Population Limits on Rapa Nui (Easter Island) before European Contact Carl P. Lipo1*, Robert J. DiNapoli2 and Terry L. Hunt3 Front. Ecol. Evol., 14 March 2018. https://doi.org/10.3389/fevo.2018.00025

Magny, M. (2006). Holocene fluctuations of lake levels in west-central Europe: Methods of reconstruction, regional pattern, palaeoclimatic significance and forcing factors.

Mann, D., Edwards, J., Chase, J., Beck, W., Reanier, R., Mass, M., Finney, B., and Loret, J. (2008). Drought, vegetation change, and human history on Rapa Nui (Isla de Pascua, Easter Island). Quaternary Research, 69(01), 16–28. https://doi.org/10.1016/j.yqres.2007.10.009

Martin, S. T. (2005). Precipitation and dissolution of iron and manganese oxides. Environmental Catalysis, 1, 61–82.

Metraux, A. 1940. Ethnology of Easter Island. Honolulu: Bishop Museum Press.

Metraux, A. 1957. Easter Island; a stone-age civilization of the Pacific. New York: Oxford University Press.

Millero, F. J. (1974). The physical chemistry of seawater. Annual Review of Earth and Planetary Sciences, 2(1), 101–150.

Murray, J. W. (1987). Mechanisms controlling the distribution of trace elements in oceans and lakes. ACS Publications.

Naeher, S., Gilli, A., North, R. P., Hamann, Y., and Schubert, C. J. (2013). Tracing bottom water oxygenation with sedimentary Mn/Fe ratios in Lake Zurich, Switzerland. Chemical Geology, 352, 125–133.

Nelson, B. J., Wood, S. A., and Osiensky, J. L. (2003). Partitioning of REE between solution and particulate matter in natural waters: A filtration study. Journal of Solid State Chemistry, 171(1–2), 51–56.

Norton, S. A. (1992). Salt consumption in ancient Polynesia. Perspectives in biology and medicine, 35(2), 160-181.

Nunn, P. D. (2007). Climate, Environment, and Society in the Pacific during the Last Millennium. Elsevier.

Peinado-Guevara, H., Green-Ruíz, C., Herrera-Barrientos, J., Escolero-Fuentes, O., Delgado-Rodríguez, O., Belmonte-Jiménez, S., andLadrón de Guevara, M. (2012). Relationship between chloride concentration and electrical conductivity in groundwater and its estimation from vertical electrical soundings (VESs) in Guasave, Sinaloa, Mexico. Ciencia e investigaciónagraria, 39(1), 229–239. https://doi.org/10.4067/S0718-16202012000100020

Puleston, C. O., Ladefoged, T. N., Haoa, S., Chadwick, O. A., Vitousek, P. M., and Stevenson, C. M. (2017). Rain, Sun, Soil, and Sweat: A Consideration of Population Limits on Rapa Nui (Easter Island) before European Contact. Frontiers in Ecology and Evolution, 5. https://doi.org/10.3389/fevo.2017.00069

Quirós, R. (2003). The relationship between nitrate and ammonia concentrations in the pelagic zone of lakes. 14.

Richards R (2008) Easter Island 1793 to 1861: observations by early visitors before the slave raids. Easter Island Foundation, Los Osos, CA, 144 pp.

Rudnick, R. L., and Gao, S. (2014). 4.1—Composition of the Continental Crust. In H. D. Holland & K. K. Turekian (Eds.), Treatise on Geochemistry (Second Edition) (pp. 1–51). Elsevier. https://doi.org/10.1016/B978-0-08-095975-7.00301-6

Rull, V., Cañellas-Boltà, N., Sáez, A., Giralt, S., Pla, S., and Margalef, O. (2010). Paleoecology of Easter Island: Evidence and uncertainties. Earth-Science Reviews, 99(1–2), 50–60. https://doi.org/10.1016/j.earscirev.2010.02.003

Rull, V. (2016). Natural and anthropogenic drivers of cultural change on Easter Island: Review and new insights. Quaternary Science Reviews, 150, 31–41. https://doi.org/10.1016/j.quascirev.2016.08.015

Rull, V. 2016b. The EIRA database: Last Glacial and Holocene radiocarbon ages from Easter Island's sedimentary records. Front. Ecol. Evol. 4, 44 http://dx.doi.org/10.3389/fevo.2016.00044

Rull, V., Montoya, E., Seco, I., Cañellas-Boltà, N., Giralt, S., Margalef, O., Pla-Rabes, S., D'Andrea, W., Bradley, R., and Sáez, A. (2018). CLAFS, a Holistic Climatic-Ecological-Anthropogenic Hypothesis on Easter Island's Deforestation and Cultural Change: Proposals and Testing Prospects. Frontiers in Ecology and Evolution, 6. https://doi.org/10.3389/fevo.2018.00032

Saez, A., Giralt, S., Valero-Garcés, B., Moreno, A., Bao, R., Pueyo, J. J., and Hernández, A. (2009). Glacial to Holocene climate changes in Easter Island (SE Pacific, 27oS). Quaternary Science Reviews, 2743–2759.

Schaller, T., Christoph Moor, H., and Wehrli, B. (1997). Sedimentary profiles of Fe, Mn, V, Cr, As and Mo as indicators of benthic redox conditions in Baldeggersee. Aquatic Sciences, 59(4), 345–361. https://doi.org/10.1007/BF02522363

Schindler, D. W. (2006). Recent advances in the understanding and management of eutrophication. Limnology and Oceanography, 51(1part2), 356–363. https://doi.org/10.4319/lo.2006.51.1_part_2.0356

Tesoriero, A. J., and Pankow, J. F. (1996). Solid solution partitioning of Sr2+, Ba2+, and Cd2+ to calcite. Geochimica et Cosmochimica Acta, 60(6), 1053–1063. https://doi.org/10.1016/0016-7037(95)00449-1

Tsay, A., Zajacz, Z., and Sanchez-Valle, C. (2014). Efficient mobilization and fractionation of rare-earth elements by aqueous fluids upon slab dehydration. Earth and Planetary Science Letters, 398, 101–112. https://doi.org/10.1016/j.epsl.2014.04.042

Vegas-Vilarrúbia, T., Corella, J. P., Pérez-Zanón, N., Buchaca, T., Trapote, M. C., López, P., Sigró, J., and Rull, V. (2018). Historical shifts in oxygenation regime as recorded in the laminated sediments of lake Montcortès (Central Pyrenees) support hypoxia as a continental-scale phenomenon. Science of The Total Environment, 612, 1577–1592. https://doi.org/10.1016/j.scitotenv.2017.08.148

Vezzoli, L. and Acocella, V. (2009). Easter Island, SE Pacific: An end-member type of hotspot volcanism. Geological Society of America Bulletin. 121. 869-886. 10.1130/B26470.1.

Wehrli, B., and Stumm, W. (1989). Vanadyl in natural waters: Adsorption and hydrolysis promote oxygenation. Geochimica et Cosmochimica Acta, 53(1), 69–77. https://doi.org/10.1016/0016-7037(89)90273-1

WHO (2003) Chloride in drinking-water. Background document for preparation of WHO Guidelines for drinking-water quality. Geneva, World Health Organization (WHO/SDE/WSH/03.04/3).

Wright H.E. (1967). A square-rod piston sampler for lake sediments. Journal of Sedimentary Research, 37 (3), 975-976.

Xiong, Z., Li, T., Algeo, T., Chang, F., Yin, X., and Xu, Z. (2012). Rare earth element geochemistry of laminated diatom mats from tropical West Pacific: Evidence for more reducing bottom waters and higher primary productivity during the Last Glacial Maximum. Chemical Geology, 296–297, 103–118. https://doi.org/10.1016/j.chemgeo.2011.12.012

Żarczyński, M., Wacnik, A., and Tylmann, W. (2019). Tracing lake mixing and oxygenation regime using the Fe/Mn ratio in varved sediments: 2000 year-long record of human-induced changes from Lake Żabińskie (NE Poland). Science of The Total Environment, 657, 585–596. https://doi.org/10.1016/j.scitotenv.2018.12.078

Figure_1 Click here to access/download;Figure;Figure_1.tif

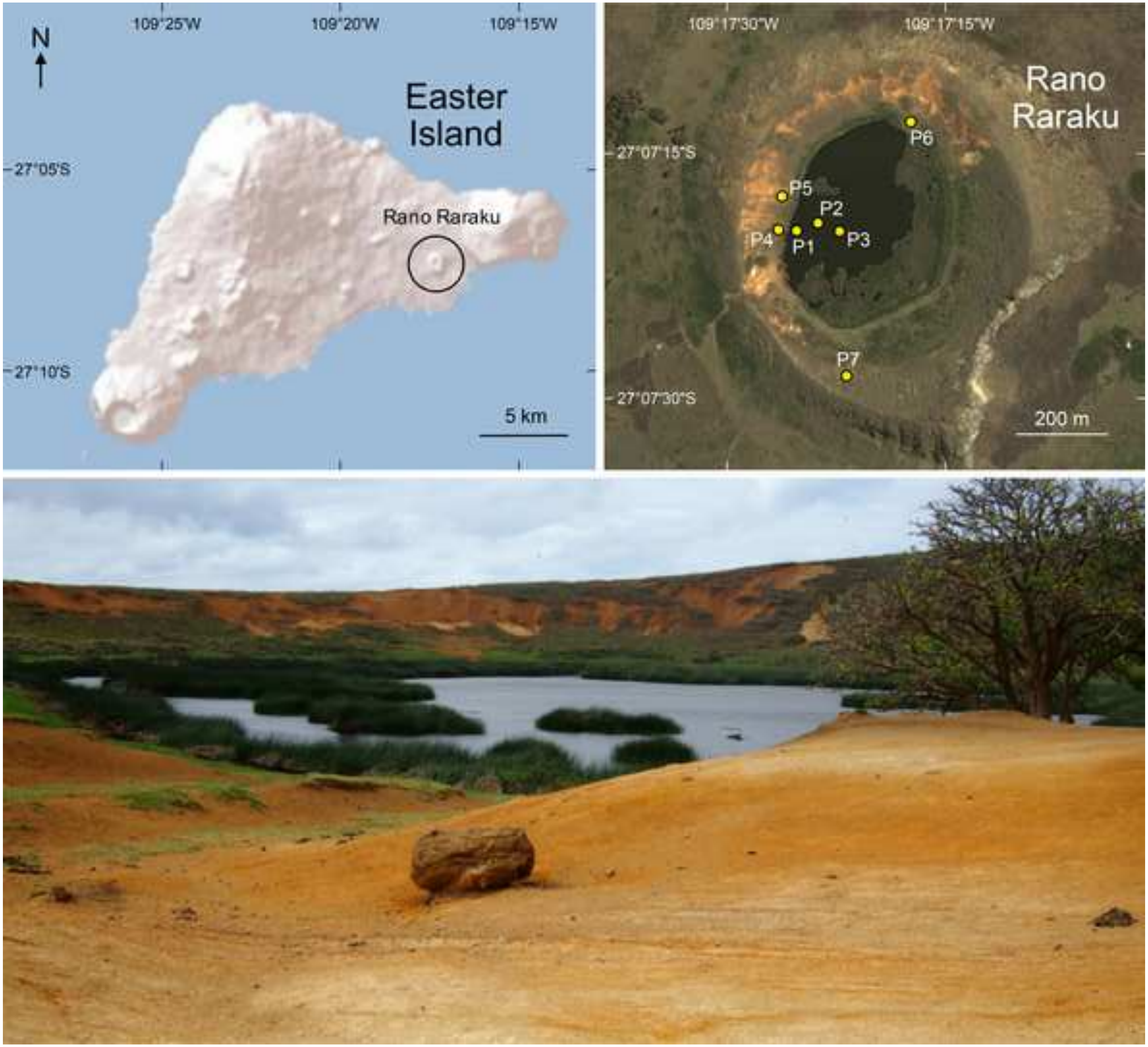

Figure_1_v2-01.jpg

Figure_2

Click here to access/download;Figure;Figure_2.tif

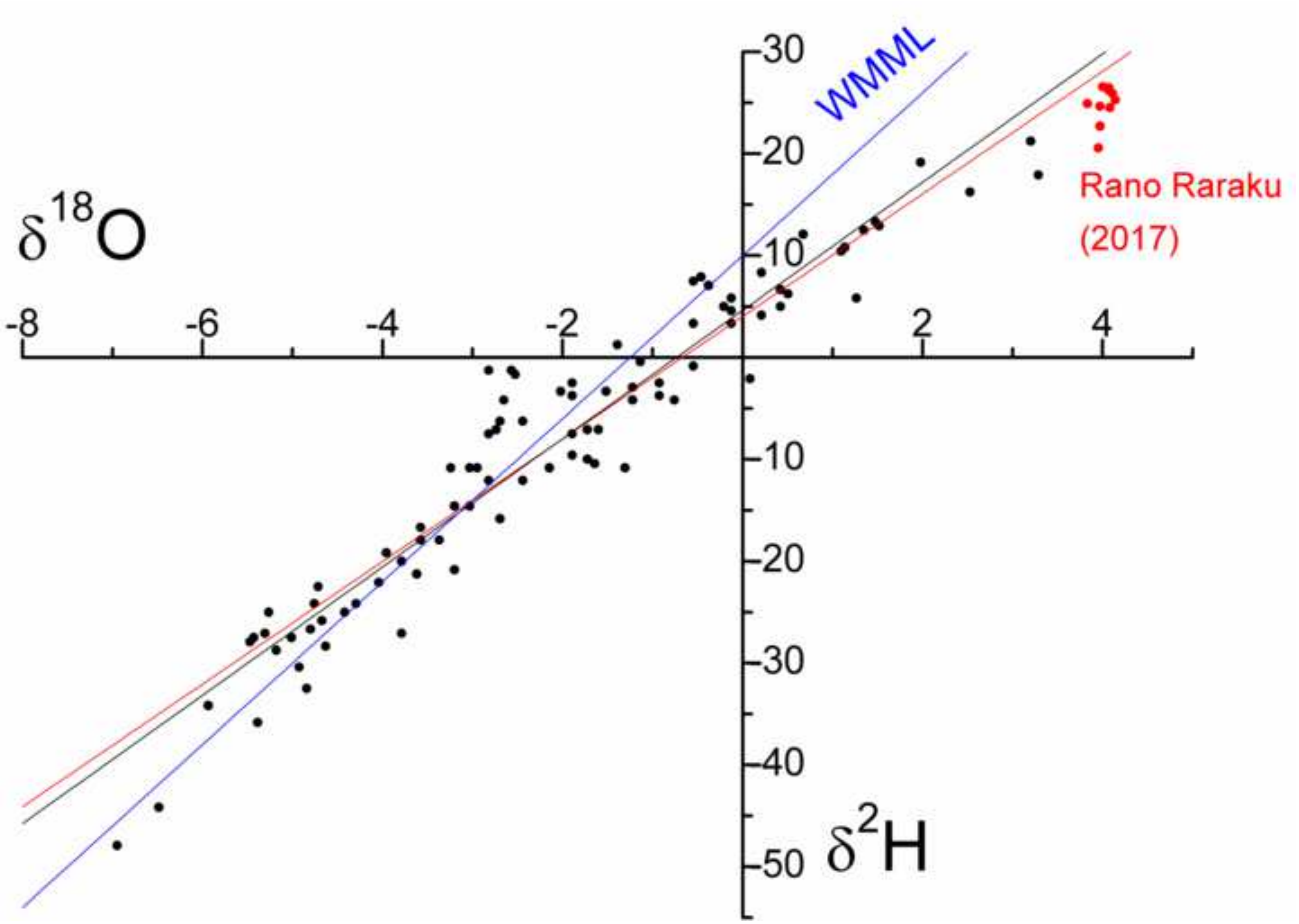

Figure_3

Click here to access/download;Figure;Figure_3.tif

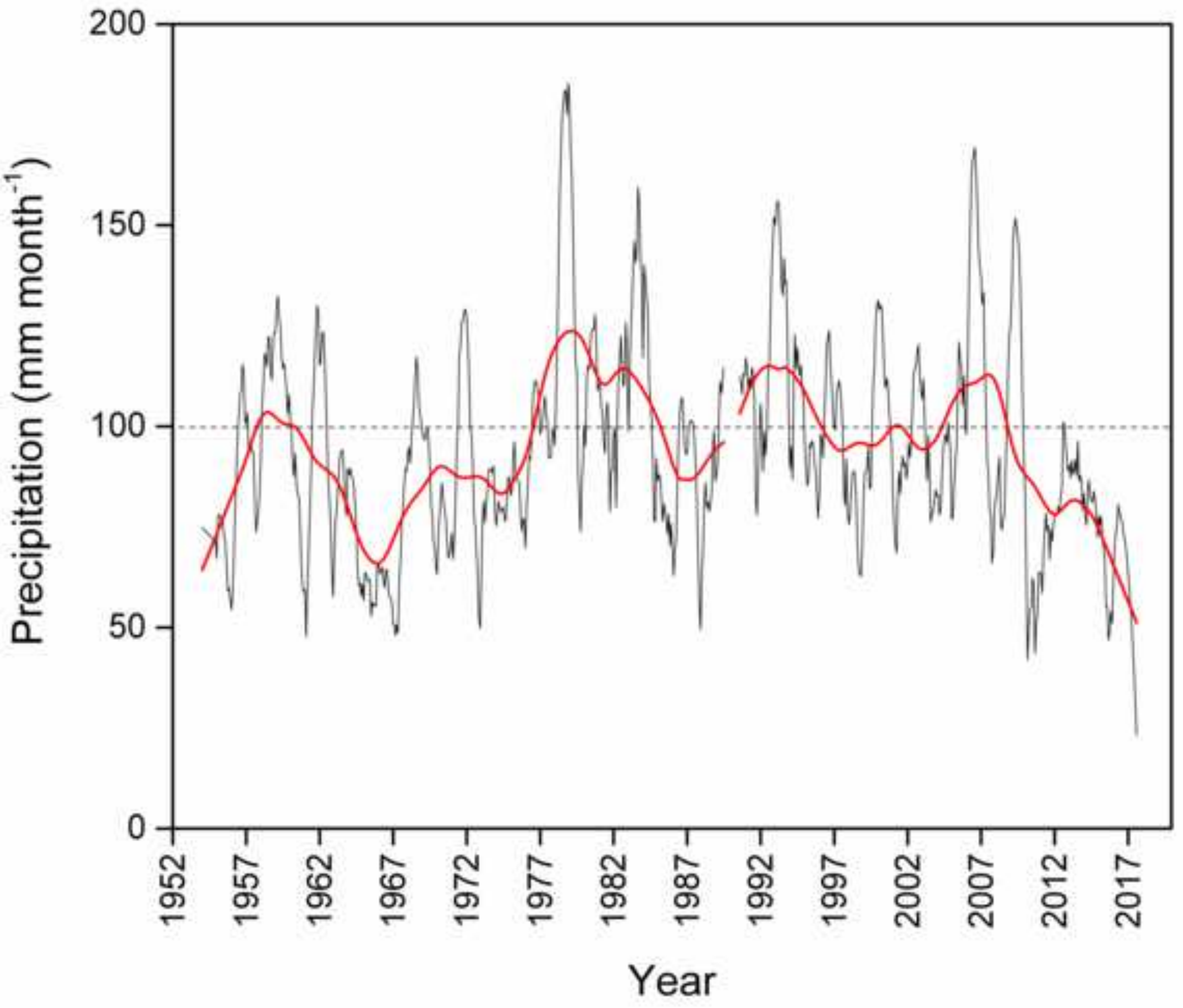

Figure_4

Click here to access/download;Figure;Figure_4.tif

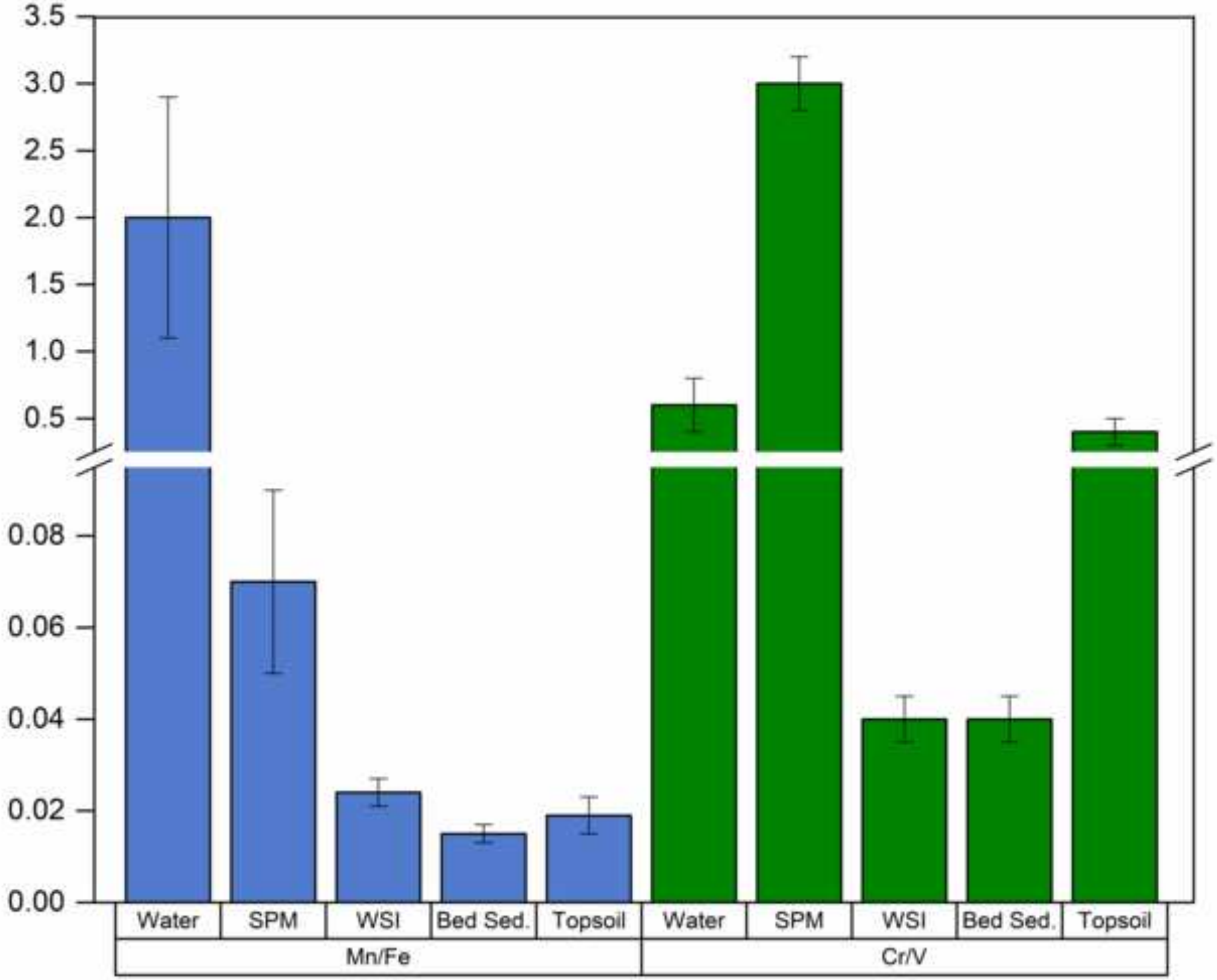

Figure_5

Click here to access/download;Figure;Figure_5.tif

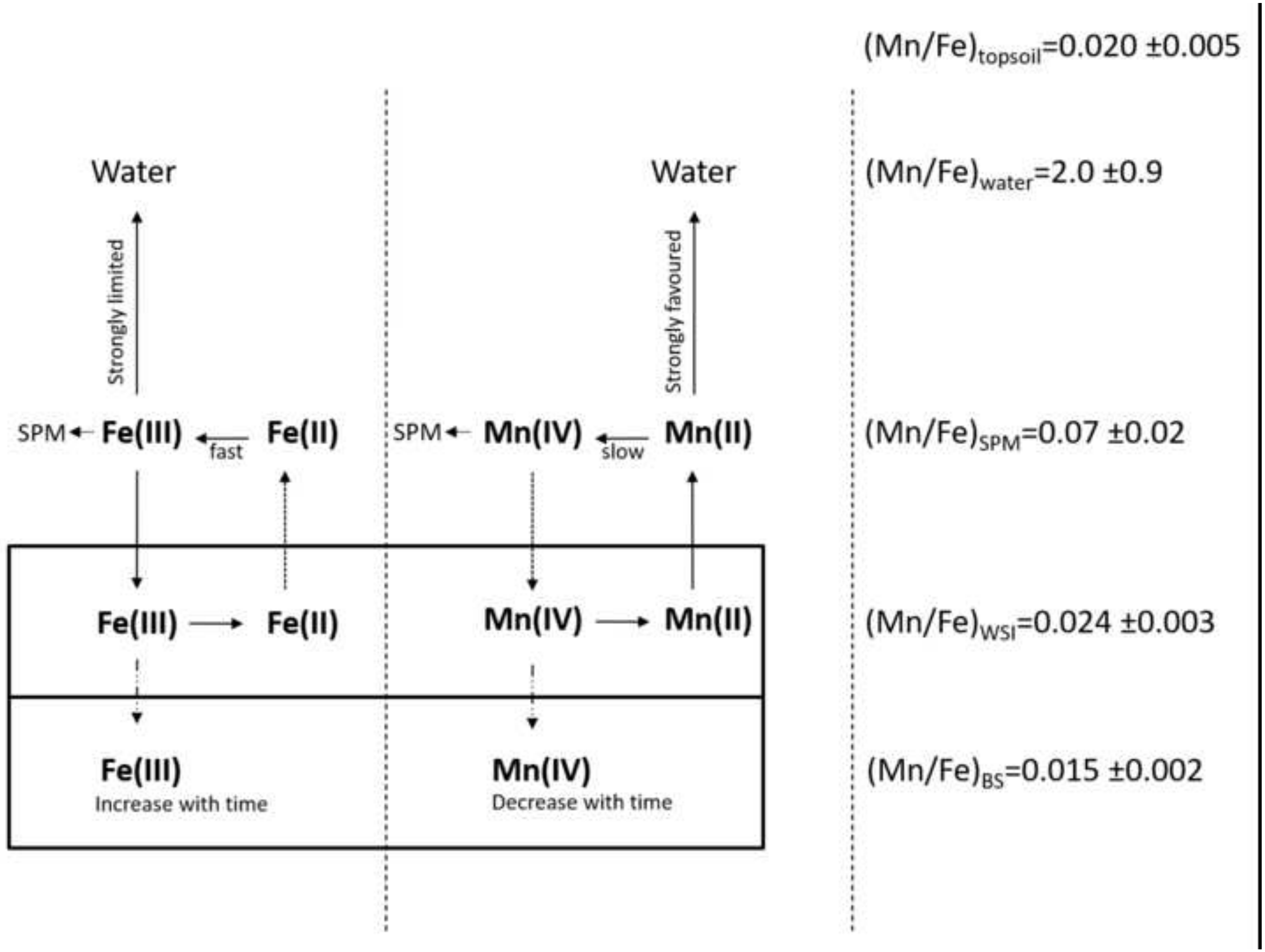

Click here to access/download;Figure;Figure_6.tif

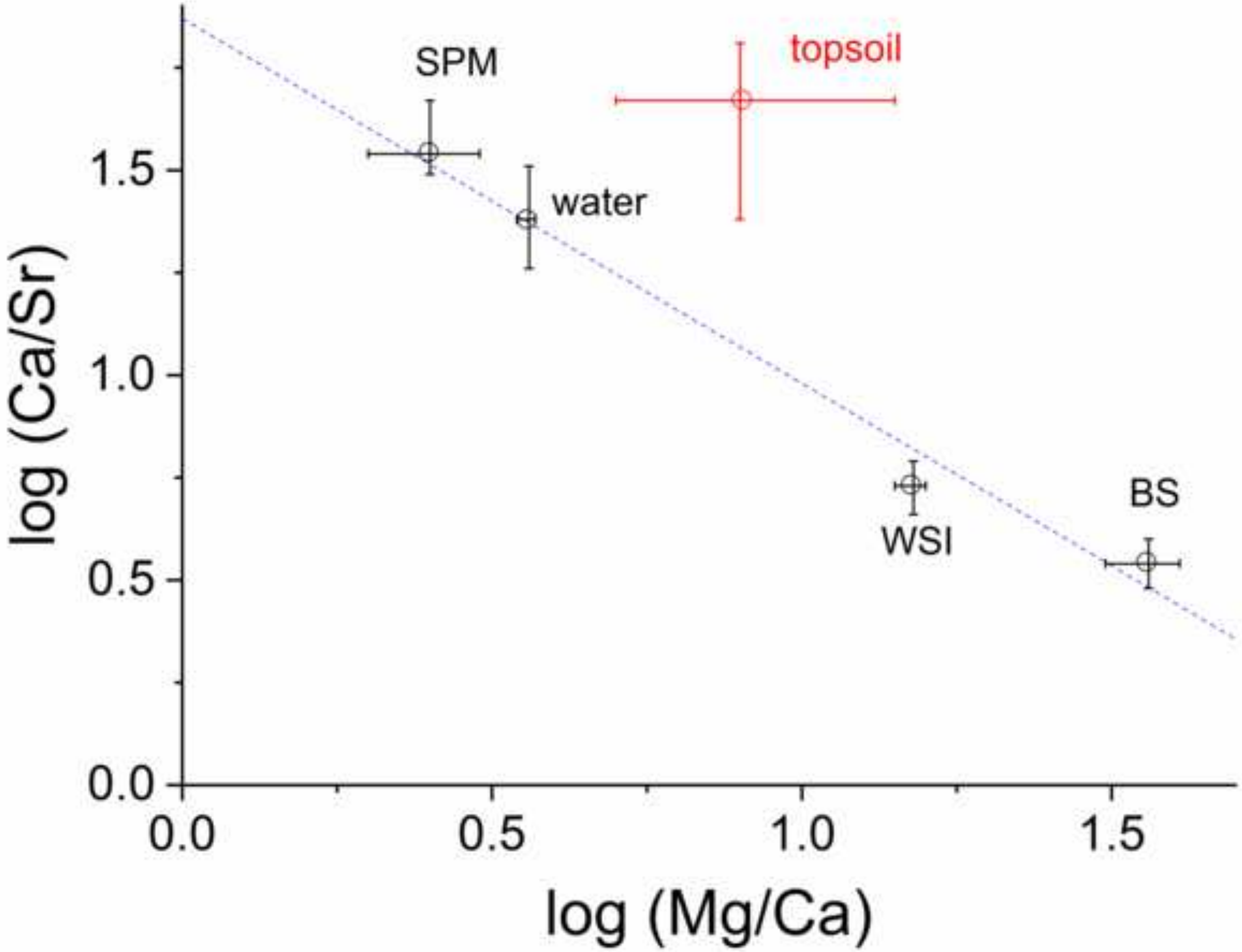

Figure_7

Click here to access/download;Figure;Figure_7.tif

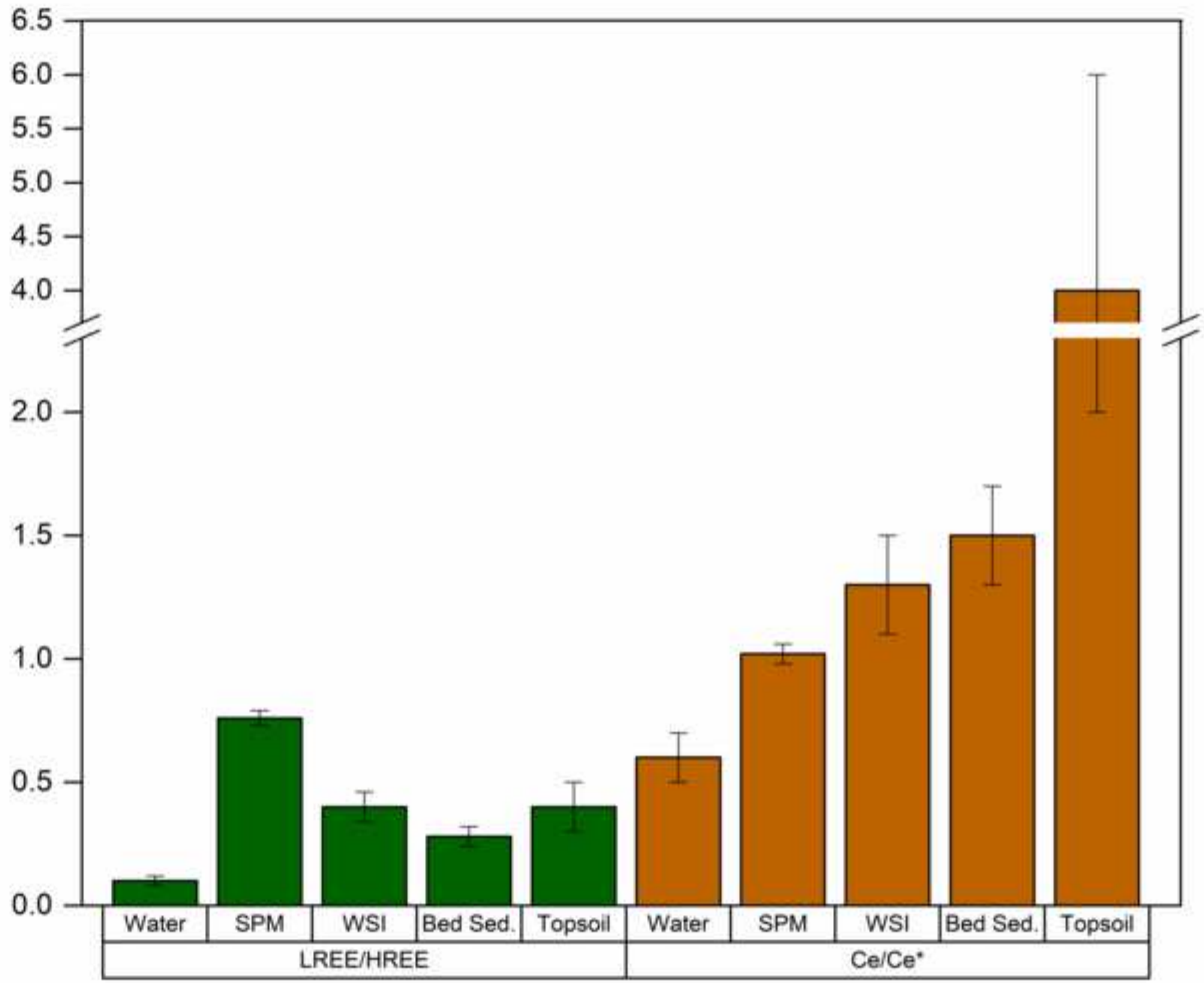

Figure_8

Click here to access/download;Figure;Figure_8.tif

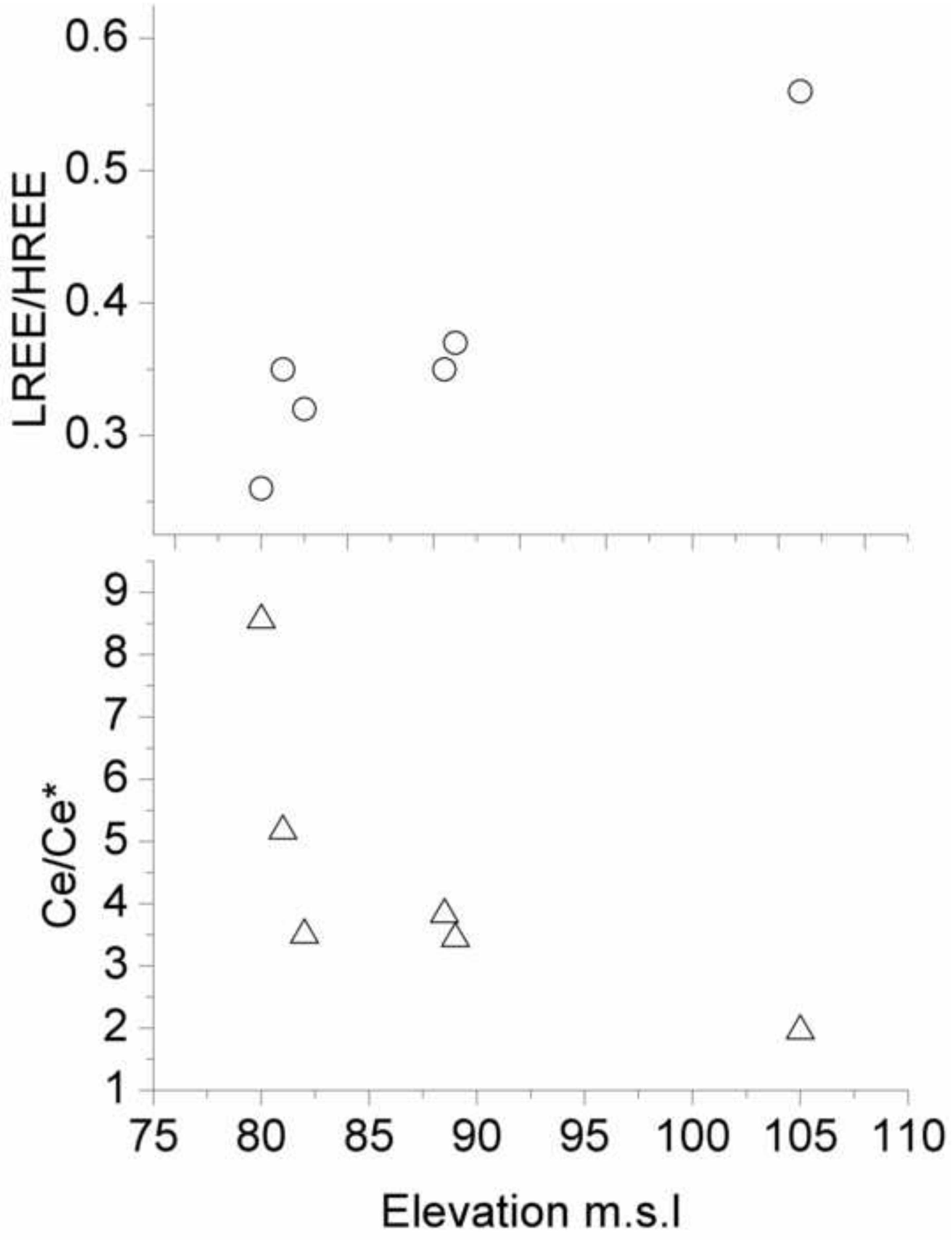

Click here to access/download
Supporting Information
Supporting-Information.docx
